# Brain Organoid Transplantation: A Comprehensive Guide to the Latest Advances and Practical Applications—A Systematic Review

**DOI:** 10.3390/cells14141074

**Published:** 2025-07-14

**Authors:** Yu-Ping Shen, Zaal Kokaia

**Affiliations:** 1 Laboratory of Stem Cells and Restorative Neurology, Lund Stem Cell Center, Lund University, 22184 Lund, Sweden; yu-ping.shen@med.lu.se; 2 Department of Physical Medicine and Rehabilitation, Tri-Service General Hospital, School of Medicine, National Defense Medical Center, Taipei 114, Taiwan

**Keywords:** brain organoids, transplantation, stem cells, neuroscience, regeneration, disease modeling, organ-on-a-chip

## Abstract

Brain organoid technology has seen significant development in recent years. This self-organized, three-dimensional, organ-oriented brain tissue model can recapitulate the process of neurogenesis and consists of diverse cell types and cellular architecture. Transplanting brain organoids in vivo could be a potential tool from bench to clinical research and has been studied for many purposes. To investigate and summarize the methodology, findings, and applications of this novel technique from current evidence, we conducted this systematic review by searching PubMed and the Embase databases for the literature ranging from 2013 to 2024. A total of 480 articles were identified, and 24 of them met the inclusion criteria. The results revealed that brain organoid transplantation had promising graft survival, neural proliferation, differentiation, and maturation, axonal growth, and functional integration into the host neuronal circuit, and has been applied to multiple applications, such as therapeutic usage, cell study platforms, and disease modeling. However, heterogeneity among studies, some significant challenges, and ethical issues remain to be considered. This comprehensive review will provide an update of what is known about this powerful, innovative method and discuss some practical aspects for future research.

## 1. Background

An organoid is a self-organized, three-dimensional, organ-oriented tissue model that can be generated from pluripotent stem cells (PSCs) [1]. The 3D brain organoid technology has been developed in recent years. The brain organoid can recapitulate the neurogenesis process, making it an ideal model for neurodevelopmental research. Its 3D structure, composition of different cell types, and organ functions provide accessible approaches to study physiology, cell migration, and cell-to-cell interactions [2,3]. For translational purposes, brain organoids, like other types of organoids, can be a powerful platform for disease modeling, drug discovery, toxicity assessment, and genetic, cancer, and infection research. It raises fewer ethical issues than animal models and diminishes the use of experimental animals. Moreover, it provides human cell representation without a gap in interspecies translation [1,2]. This model allows for longitudinal observation and sustained viability, with sufficient tissue availability, instead of a fixed temporal representation and scarce resources of post-mortem material [4].

Towards more advanced applications, organoids can be used for transplantation in vivo or ex vivo. Intestine [5], liver [6], pancreas [7], kidney [8], and retina [9] organoid transplantation has been studied in several projects [1,10]. As the brain is a more complex and fragile organ with poor regenerative ability, heterogeneity, intricate tract projection, and specific region-related neural functionalities, it is even more difficult to repair damage, and focal cell or tissue replacement is challenging [11].

Human cerebral organoid (hCO) transplantation into the mouse brain was first reported in 2018, and it was shown to be a practical method [12,13]. It can provide diverse cell types, plentiful cell numbers, a well-organized structure, and better graft survival compared to cell transplantation. The potential brain-repairing strategy was then explored by more studies, mainly focusing on traumatic brain injury (TBI) and stroke in animal models [14,15,16,17]. With induced PSC technology, some problems of traditional transplantation, such as graft rejection and a lack of organ donors and cell sources, can also be solved if applied to autologous transplantation in the future [18]. In addition, the studies of brain organoid transplantation are not only for therapeutic purposes but also for the study of development or disease modeling, physiology, functional integration, and vascularization [19,20,21,22]. In this article, we will review current publications regarding this innovative approach with the aim of providing a comprehensive overview of this field.

## 2. Search Strategy

**Figure 1:**
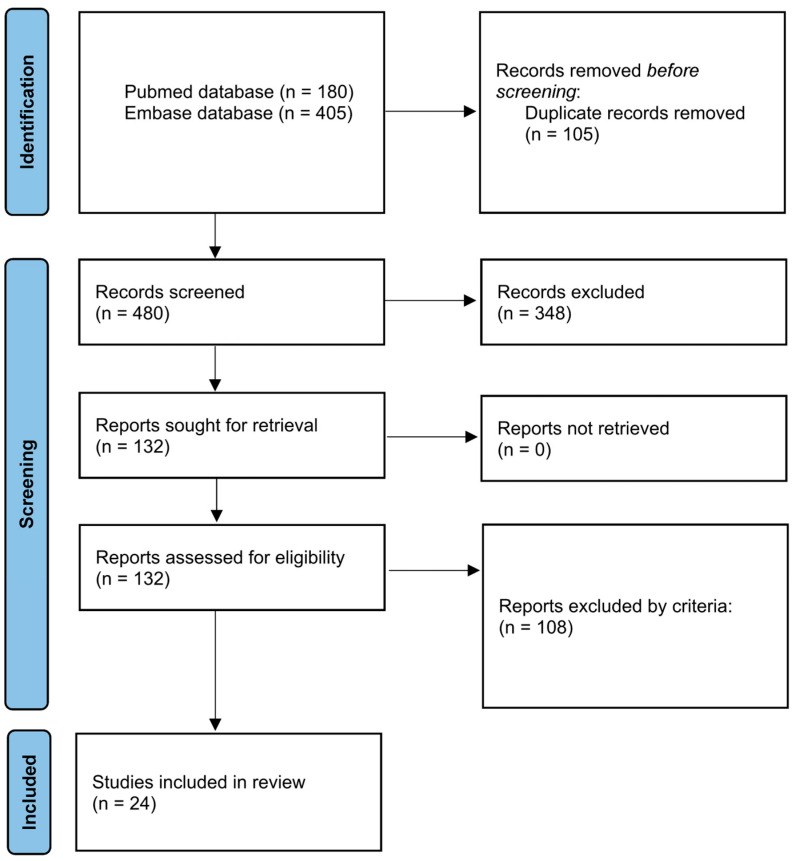
Flow diagram for new systematic reviews based on PRISMA 2020.

This systematic review was conducted following the guidelines of the Preferred Reporting Items for Systematic Reviews and Meta-Analyses (PRISMA) statement [23] (Figure 1), using the protocol registered in the International Prospective Register of Systematic Reviews (PROSPERO) (ID: CRD 1076979). PubMed and Embase were used to create the article database. The general structure of the search strategy was composed of “brain-related organoid” and “transplantation” and linked by Boolean connectors. The details of the search term setup are listed in Table 1. The time frame ranged from 2013 to 2024. In Embase, we further applied the filter by publication types that included “article”, “review”, “preprint”, and “article in press” to obtain more concise results. Duplicates were first removed by EndNote 21 and then by manual review. Articles with full-text available were screened by title and abstract.

## 3. Selection Criteria

Two independent reviewers reviewed the data. We selected articles reporting original studies containing intracerebral or intracerebellar transplantation of human brain organoids in vivo. Review articles or protocols without original results or studies that transplanted dissociated cells or used only extracranial or in vitro transplantation were excluded.

## 4. Overview

**Figure 2:**
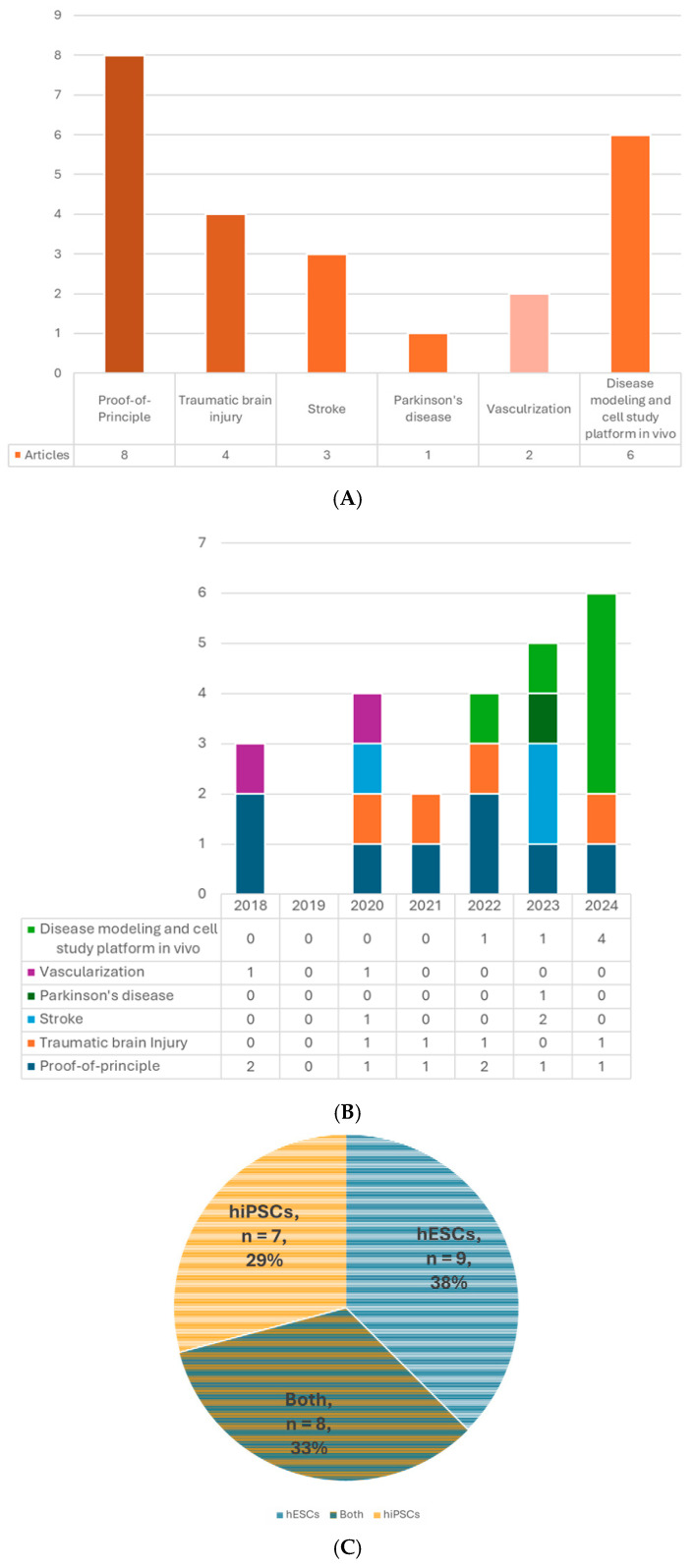

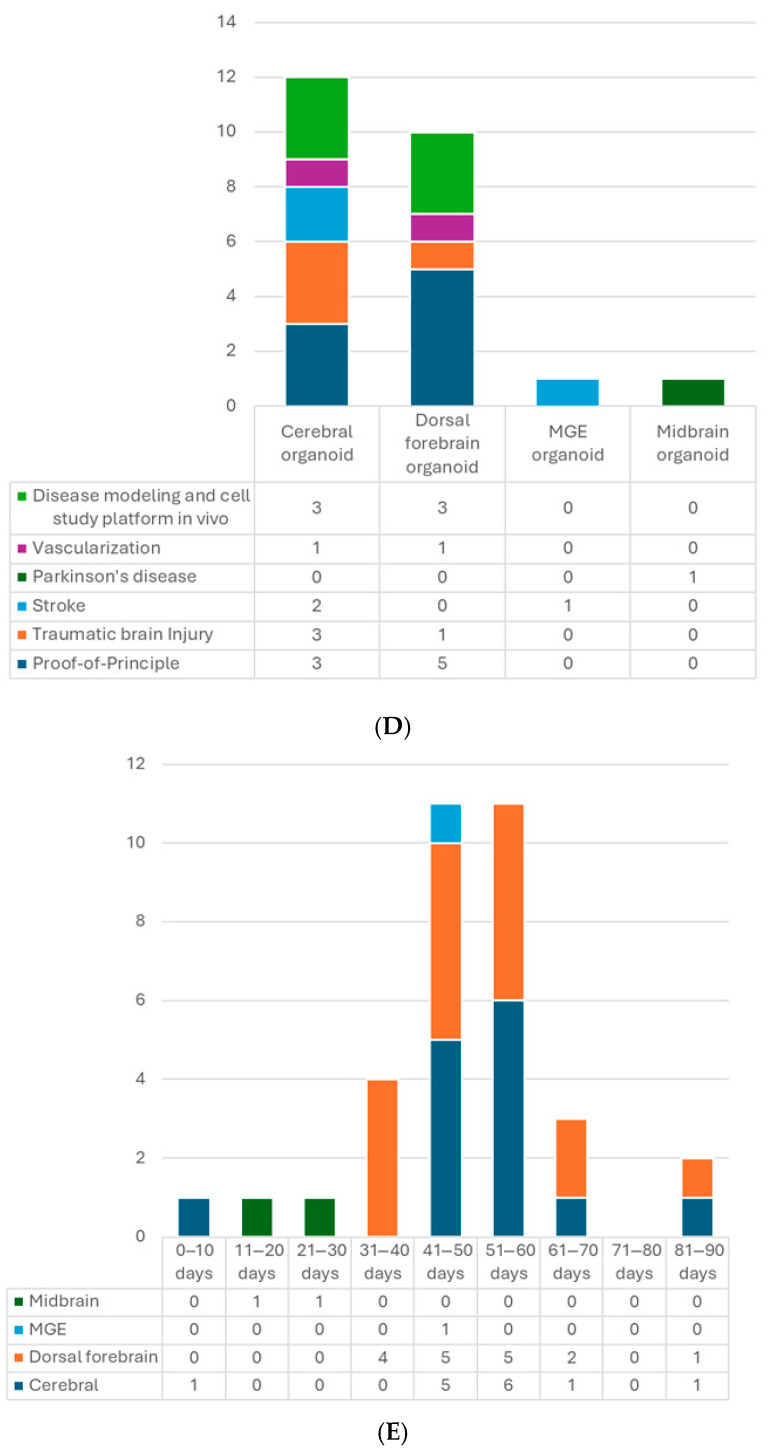

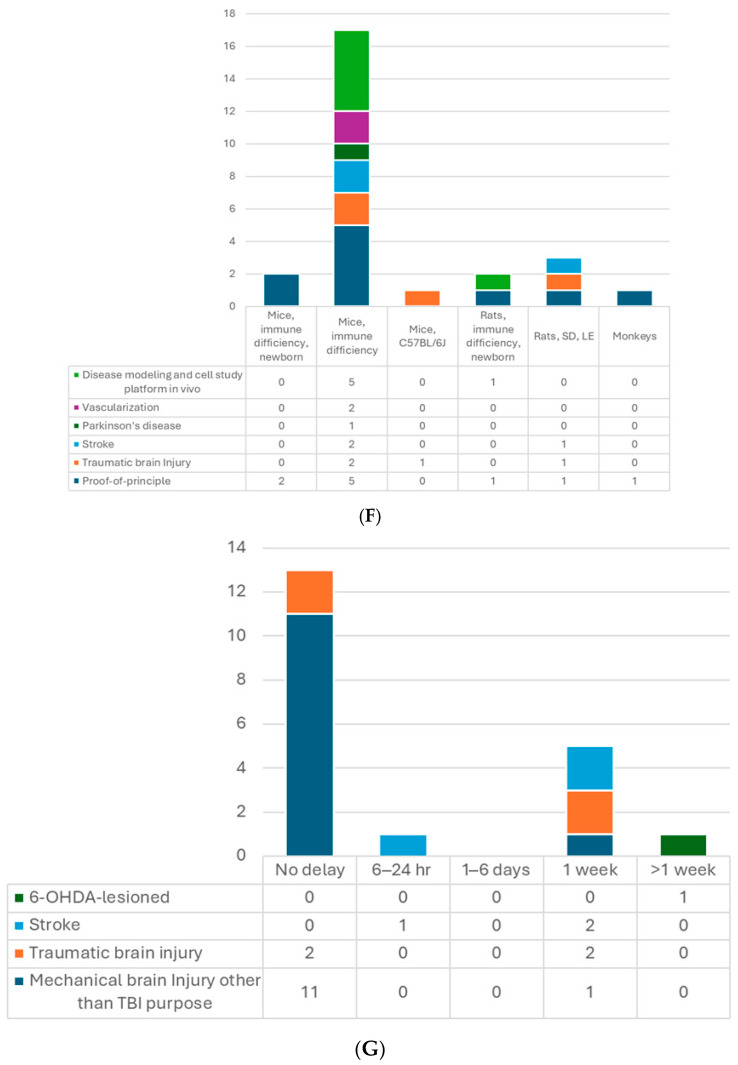

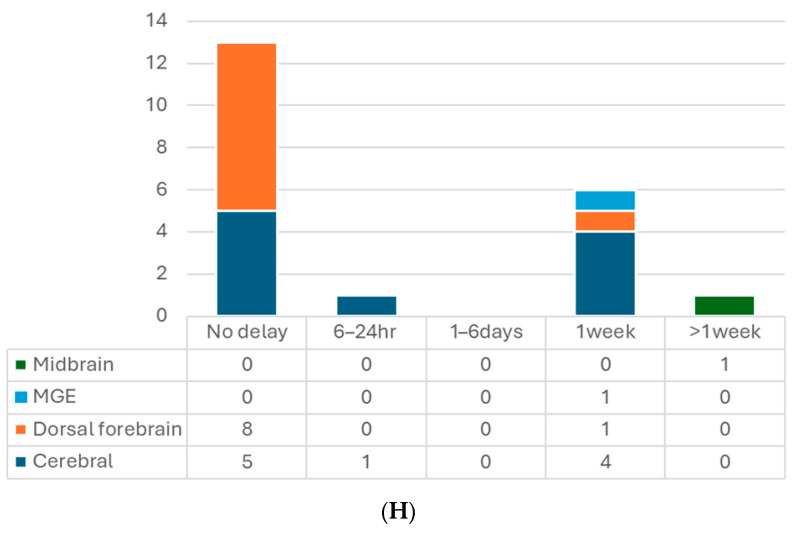
(**A**) Number of articles by research type. (**B**) Number of publications by year. (**C**) Number of studies by pluripotent stem cell type. (**D**) Number of studies by type of organoid for transplantation. (**E**) Number of studies by age of organoids at the time of transplantation. (**F**) Number of studies by animal models. (**G**) Number of studies by timing of transplantation—model. (**H**) Number of studies by timing of transplantation—organoid type. 6-OHDA, 6-hydroxydopamine; hESCs, human embryonic stem cells; hiPSCs, human-induced pluripotent stem cells; MGE, medial ganglionic eminence; TBI, traumatic brain injury.

A total of 24 published articles that met the selection criteria were included (Table 2, Figure 2A,B). The risk of bias of each included study was assessed by the SYRCLE’s risk of bias tool [24] (Table 3). The cell source, type and age of the organoids, size and cell number, recipient characteristics, the site, timing, and procedure of transplantation, outcome measures, including host and graft survival, grafted cell differentiation and maturation, axonal projection, cell migration, synaptic formation, electrophysiological neuronal activity and functional integration, vascularization, host neurogenesis, behavioral functions, current applications, etc., will be thoroughly summarized and reviewed.

## 5. Organoid

### 5.1. Cell Source 

The brain organoid can be generated from PSCs. The two major types of human PSCs are embryonic stem cells (hESCs) and human-induced pluripotent stem cells (hiPSCs). The former is acquired from the inner cell mass of the blastocyst; the latter can be obtained by gene reprogramming of the somatic cells, such as fibroblasts, blood cells, etc. [39]. These two types of cells share similar properties of self-renewal and pluripotency with little difference in gene expression, methylation, proteomic profiles, or epigenetic memory [40,41,42] and even have comparable outcomes in some therapeutic studies [43,44]. Using iPSCs can avoid many ethical concerns associated with hESCs because it does not require the destruction of human embryos and has more easily accessible sources [40]. Even more, for future clinical applications, autologous transplants are possible if the iPSCs are generated from the same individual, which could prevent graft rejection problems. Nine out of 24 studies used only hESCs to generate organoids for transplantation, 7 used hiPSCs, and 8 used both types (Table 2, Figure 2C). 

In Cao’s study, they compared the cell composition of brain organoids derived from hESC H9 and hiPSC IMR90–4 after differentiation in vitro for 50 days. They noticed similar results between these two types of organoids [17]. Dong et al. also found similar cortical differentiation patterns of these two types of cell-derived hCOs after transplantation [26]. Jgamadze et al. further investigated their dorsal forebrain organoid (hDFO) grafts generated from hESCs H9 and two iPSC lines AICS and C1.2. They observed similar results from hESCs and iPSCs, including cell apoptosis, the number of Iba1+ microglia surrounding the grafts, and the survival rates of the host animals and grafts. But some interline differences were noted. The AICS grafts were significantly smaller with fewer CD31+ (vascular endothelial marker) structures at 2 months post-transplantation (mpt), while the H9 grafts had a slightly higher number of GFAP+ astrocytes in the adjacent host brain tissue [20].

### 5.2. Types of Organoids

The first hCO and hDFO cultures in vitro were reported in 2013 by Lancaster et al. and Kadoshima et al. [45], respectively. (To note, the term “cortical organoid” is usually referred to as “dorsal forebrain organoid”. However, in some papers, it could mean “cerebral organoid”). Many protocols have been developed in the following years. Non-guided protocols utilize the characteristics of self-organization and cell-to-cell interactions to generate hCOs without using patterning growth factors [46]. Guided protocols can generate brain-region-specific organoids by modulating small molecules [4,47]. Nowadays, organoids of the forebrain (including cerebrum [46], dorsal [45] and ventral [48,49] forebrain, striatum [50], ganglionic eminence (GE) [51], choroid plexus [52], hippocampus [52], thalamus [53], and hypothalamus [54]), midbrain [55], and hindbrain (including cerebellum [56] and brain stem [57]) could be yielded in vitro. In brief, dual-SMAD inhibition, inhibiting bone morphogenic proteins (BMPs) and TGFβ pathways, can differentiate the stem cells into neuroectoderm. TGFβ and WNT inhibitors could be used to drive toward telencephalic fate [47]. Dual-SMAD inhibition followed by fibroblast growth factor (FGF) 2 and epidermal growth factor (EGF) is a commonly used recipe for generating hDFOs [58]. Inhibiting the Sonic Hedgehog (SHH) pathway will lead to dorsal cortical differentiation [48,59]. In contrast, SHH activation will guide ventral forebrain patterning to generate medial GE [48,49]. Striatal organoids from lateral GE can be obtained by inhibiting WNT by activating TGFβ and retinoic acid (RA) [50]. Both thalamus and hypothalamus organoids are initiated with dual-SMAD inhibition, with additional insulin, a MEK-ERK inhibitor, and BMP7 for the former [53] and SHH and WNT activators for the latter [54]. To obtain the choroid plexus or hippocampal organoid, WNT and TGFβ are first inhibited to generate a telencephalon. Then, BMP is added, and the WNT pathway is reactivated with a GSK3 inhibitor [52]. To generate midbrain organoids, dual-SMAD inhibition with WNT and SHH activation is used with or without FGF8 [54,55,60]. A TGFβ inhibitor and FGF2, then FGF19 and SDF1, are utilized to form cerebellar organoids [56]. For brain stem organoid generation, dual-SMAD inhibition is also used with the presence of insulin, progesterone, and transferrin, and then bFGF is added later, followed by EGF [57].

Several types of brain organoids have been used for intracerebral transplantation (Table 2, Figure 2D). So far, most studies implanted unguided hCOs followed by those transplanting hDFOs, possibly because the main target of interest and a more straightforward approach is the cortex, and the widely used dorsal forebrain-guided protocol came out later than the unguided one [58]. Unguided hCOs generally display a dorsal forebrain identity but are not as “pure” as the hDFOs. They could contain cells from other brain regions, such as the retina or hippocampus [4,46]. Because the current hCO and hDFO models lack endothelial cells (ECs) [61,62], some studies integrated stem cell-derived ECs into the brain organoid culture and successfully built their vascular connection with the host [21,22]. In addition, since the microglia are absent in the hDFO model and the mature glial cells only appear in the late stage (around eighty to a hundred days of differentiation) [4], Gage’s group also developed neuroimmune hDFOs and glia-enriched hCOs and transplanted them into mice brains as in vivo platforms for studying microglial and astrocyte cells, respectively [34,35]. Other than dorsal forebrain-like organoids, one study implanted medial GE organoids (hMGOs), which are more like ventral forebrain identity, in the stroke-damaged cortex, aiming to utilize GABAergic interneurons to modulate the brain and facilitate recovery [31]. Midbrain organoid (hMO) transplantation has also caught attention because of Parkinson’s disease. Zheng et al. showed successful establishment of bidirectional connections between hMOs and host striatum in Parkinson’s model and improvement in motor function [32]. Studies of transplanting assembloids, which merge two or more organoids, are also undergoing.

### 5.3. Age of Organoids at the Time of Transplantation 

Organoids are developing tissue. The structure, cell composition, and maturity will change with time. Since these features may influence the result of transplantation, the age of organoids at the time of transplantation is crucial. The developing timeline will also depend on the type of organoid and the protocol. For example, in the hDFO scenario, the transcriptomes at 52 DIV and 76 DIV stages resemble those of human mid-fetal prenatal brains (10–19 and 19–24 post-conception weeks, respectively) [58]. And they could reach postnatal stages after 250 DIV [63]. Most hCO and hDFO transplantations were conducted with 40–60 days in vitro (DIV) organoids (Figure 2E). Glial cells, forebrain precursor cells, and some mature neurons could be seen at this stage [4]. Senior organoids up to 88 DIV have also been used, which have more mature and differentiated neurons and more segregation of upper- and lower-layer cortical neurons with less proliferation activity [20]. In addition, 7 DIV premature organoids have been transplanted. At this stage, only Nestin+ neural stem cells (NSCs) are present without advanced differentiation [33].

Wang et al. compared 55 DIV with 85 DIV hCOs and revealed higher cell numbers, more neurons, and astrocytes in the latter ones with fewer neural progenitor cells. After transplantation into an in vivo environment, 55 DIV organoids showed a better effect in promoting neurogenesis and greater cell survival [14]. Another study compared 6-week and 10-week-hDFOs. Dorsal forebrain progenitor marker PAX6, neuronal marker MAP2, radial glial-like cells with proliferation marker Ki67, and sub-cerebral projection neurons marker CTIP2 could be seen at the 6-week timepoint, but no cells were positive for the callosal projection neurons marker SATB2. Notably, 10-week-old organoids had a larger size and an increasing thickness of the total epithelium, especially in the layer of CTIP2+ cells. The percentages of PAX6+ cells and Ki67+ cells decreased while that of CTIP2+ cells increased. The SATB2+ cells showed up at this stage. After transplanting, both 6-week and 10-week organoids could provide axonal extensions along the host cortical spinal tract. And the 6-week-hDFOs could extend a larger number of axons. However, they could also result in graft overgrowth in the host brain, which was not observed in the scenario of 10-week-old organoid transplantation [25].

The only study of transplanted hMGEOs revealed a strong expression of MGE marker NKX2.1 and downregulation of the PAX6 of the organoids on 30 DIV, suggesting a ventral fate. On 65 DIV, GABAergic interneuron markers GABA and GABA synthesizing enzyme GAD67 were widely seen in hMGEOs, as well as subtype markers of GABAergic interneurons, including calbindin, calretinin, somatostatin, and neuronal nitric oxide synthase. They also disclosed mature neuron patterns by electrophysiological analysis and calcium imaging on 45 DIV. In this case, they finally chose 50 DIV organoids for transplantation.

Regarding the hMOs, Zheng et al. examined mRNA expression in vitro and found transcription factors FOXA2 and EN1, associated with early dopaminergic (DA) lineage development, increased on 7 DIV. NURR1 and TH expressed by post-mitotic DA cells showed up on 15 DIV and peaked by 25 DIV. Neuronal markers TUJ1 and MAP2 gradually increased from 0 to 25 DIV. Regarding transplantation, they further demonstrated that 15 DIV organoids are the better choice than 10 and 25 DIV since they not only survived but also matured into DA neurons [32]. Another study used hMOs as the cell source for transplantation and chose 30 DIV organoids to be the candidates, based on the statement with the highest proportion of DA progenitors and neurons and few oligodendrocytes and astrocytes [64]. Both studies showed that the grafts could integrate into the neural network of the PD mice and improve motor function. To be noted, the latter study only transplanted dissociated cells from hMOs.

### 5.4. Size and Cell Number

Unlike cell transplants, organoids are much bigger and have uneven sizes. They can grow up to 1 to 2 mm in diameter at the time of transplantation. For better quality control, some studies control and select the size of organoids for transplantation from around 150 to 600 μm, even with cell counting from fifty to seventy thousand cells of each cerebral or forebrain organoid (and 400,000 cells of each hMO), in addition to the age of the organoids. Normally, the number of transplanted organoids varies between one and three. In Cao’s study, they found that three 50 DIV organoids (~600 µm, 70,000 cells each) could completely fill the infarcted cavity of their photothrombotic lesion instead of one organoid [17]. To achieve more consistency and reproducible results, in six studies, the organoids were cut to achieve a uniform size and fit transplanting tools, such as a needle [25,26,28,30,32,38]. None of the reviewed studies reported adverse effects caused by mechanical injuries to organoids. Reports revealed that mechanical cutting could improve the quality of hCOs by increasing oxygenation and preventing necrotic core formation [65,66].

### 5.5. Additional Treatment of the Organoids In Vitro

To be on the safe side, antibiotics were usually added to the culture medium to prevent infection. The most common regimen is 100 U/mL of penicillin and 100 µg/mL of streptomycin.

To prevent cell death of the organoid grafts, Jgamadze et al. incubated the hDFOs in 20 µg/mL of Necrostatin-1 (Nec-1), a specific inhibitor of the kinase domain of receptor-interacting protein kinase-1, for 24 h before transplantation [20]. They achieved a robust graft survival rate of 82.1% up to 3 months. However, there was no control group without Nec-1 treatment for comparison in this study.

To lower the risk of tumor formation, Zheng et al. pretreated the 13 DIV iPSC-derived hMOs with 40 μM quercetin (QC), which targets an hPSC-specific antiapoptotic factor that leads to apoptotic cell death of PSC, for 16 h [32]. They noticed the mRNA levels of DA lineage-related factors would be affected by QC treatment for 1 day and could recover 3 days later. They further investigated engrafted hMOs (15 DIV when transplanting) and found they were negative for OCT4 and Ki67 at 6 weeks post-transplantation (wpt), indicating low tumorigenic risk. They also transplanted the same age hMOs into the subcutaneous space of severe combined immunodeficiency (SCID) mice to detect teratoma formation. No tumor or grafted cell migration was found over the whole body. Moreover, no tumor was formed following intra-striatal implantation. They suggested that the removal of undifferentiated cells reduced the tumorigenic risk [32].

To facilitate organoid development, one study used 0.2 W low-intensity ultrasound (LIUS) to treat hDFOs from 18 DIV. They found that LIUS stimulation could improve the proliferation of neural progenitor cells (NPCs) and reduce apoptosis during 35–75 DIV of follow-up. They also noticed that LIUS stimulation could delay neuronal differentiation at early stages with more SOX2+ progenitors and fewer MAP2+ mature neurons on 35 DIV but facilitate the differentiation at the later stages with an increase in cortical plate thickness on 55 and 75 DIV. Synaptic density and electrophysiological activity, including average firing rates, the number of bursts, and average burst durations, also increased. The transcriptional profiles showed an enriched expression of forebrain development, neurogenesis, axon genesis and guidance, WNT signaling, and responses to mechanical stimuli in LIUS-treated organoids. Moreover, they transplanted 50 DIV LIUS-treated organoids into the S1 cortex of mice and assessed the outcome at 2 and 5 mpt. Compared to the control organoid-grafted group, the LIUS-pretreated group had progressive blood vessel growth and neuronal differentiation with decreased glial differentiation. Higher synaptic connectivity with host neurons and a higher density of long-distance axonal projections from the grafts to distant brain targets were also observed [38]. The same group also investigated the effects of electrical stimulation (ES) on the hDFOs. They conducted ES with 400 mV, 250 Hz, 100 pulses per train (0.4 s stimuli, 0.6 s rest), 4 min per day during 18–26 DIV (with or without 35–42 DIV). The neuronal layer thickness increased on 55 DIV, and more CTIP2+ subcortical projection neurons were found on 75 DIV in the ES groups, as well as an increase in synaptic structure on 120 DIV. Enhanced neuronal connectivity was also noted by calcium imaging on 120 DIV. Upregulation of the genes related to neurodevelopment was found at 35 DIV. The ES-treated organoids were able to mature after being transplanted for 2–4 months, with longer total neuronal length and more vascularization than the control group. More and longer axonal projections were also seen [28]. These findings indicate that the LIUS and ES modalities could facilitate organoid development both in vitro and in vivo. The mechanisms behind these effects of LIUS and ES were related to YAP, a mechanosensitive transcriptional activator, and the CAMKII-PKA-CREB pathway, respectively. No tumor formation was reported in these studies [28,38]. These researchers further created a more advanced organoid–brain–computer interface (OBCI) model, which inserted an electrode into the organoid to promote neurodevelopment and functional maturation. However, these OBCI-pretreated organoids were not implanted in this study. Instead, they built an in vivo OBCI model, which is mentioned below [30].

### 5.6. Brain Organoids for Transplantation

In general, the quality of brain organoids can be assessed by the following criteria: the clearing of embryoid body borders, the formation of radially organized neuroepithelium, and the outgrowth and development of defined buds, displaying an appropriate differentiation/maturation phenotype without massive cyst formation or premature differentiation [12,13,67,68]. The organoids that pass these criteria could be the proper candidates for transplantation.

## 6. Recipient

So far, the most used model for transplantation has been the mouse (Table 2, Figure 2F). In total, 18 out of the 24 included studies used the mice, and the dominant strain was SCID mice, with only 1 study using CD1 [13] and another one using the C57BL/6J [29] strain. Up to 17 studies transplanted used 4- to 12-week-old mice, while two studies included P7−10 newborns [13,25]. Kitahara’s study used 7-day-old and 6-week-old mice with similar settings. They obtained comparable results regarding axon extension and graft growth patterns [25]. There are five studies using rat models with Sprague–Dawley [14,16], Long Evans [20], or athymic (FOXN^−/−^) rats [19,36]. The athymic ones were P3−7 newborns, in which the thalamocortical and corticocortical axonal projections have not yet been completed [69], and the other adults’ weight ranged from 220 to 300 g. One protocol stated that neonatal animals have a more plastic host environment, which could improve human–host circuit integration. They also compared mice and rat models for the brain organoid transplantation and suggested that using rats may have less harmful impacts on animal functioning and could achieve more complex behavioral readouts, whereas, the mouse models have an advantage in genetic modification for disease modeling and molecular labeling of cells [70]. Regarding sex, male and female rodents were both used; however, male animals were more favored in TBI or stroke studies. That may be because young females have a protective advantage in behavioral outcomes and pathology resulting from female sex hormones [71,72]. There was only one study transplanting hDFOs into non-human primate brains, which used 3-year-old cynomolgus monkeys [25]. They observed that grafts survived in the cerebral cortex of monkeys and were able to provide axonal extensions along the callosal and sub-cerebral projections. Additionally, in theory, immunosuppression is required in transplantation studies with immunocompetent animals. The limited period of immunosuppressant usage may limit the follow-up time, which should be considered [20]. There is still one study using CD1 immunocompetent mice without immunosuppression [13]. The authors claimed that immunosuppression was only mandatory to achieve engraftment beyond 2 months [13,73]. Their results demonstrated the feasibility within 4 weeks of follow-up in the mouse model. In addition, immunosuppressants have never been reported to negatively affect the motor function recovery in NPC transplantation for the spinal cord injury model [74]. However, this has not been investigated in a brain organoid transplantation study.

## 7. Transplantation

### 7.1. Site of Transplantation

The neurological functions of the brain vary across regions. Hence, the site for transplantation should be selected properly. To date, the main chosen location for transplantation is the cortex, which contains multiple neurologic functions and is easily accessible. The target areas are distributed from the medial prefrontal cortex (PFC), frontoparietal cortex (S1 and motor), and retrosplenial cortex to the visual cortex (Table 2). The related functions include higher cognitive functions and emotions, sensorimotor, locomotion and spatial cognition, and visual function. A dense vascular network is located below the transplanted area, such as the retrosplenial cortex, which may provide better support to the grafts [12]. In addition to the cortex, organoid transplantation has also been performed on the striatum [32,33] and hippocampus [37] in studies regarding Parkinson’s disease and cell fate regulation by the brain region. The effects of organoid transplantation are discussed below.

### 7.2. The Timing of Transplantation

When it comes to transplantation in models of human diseases, timing is an important factor. In most of the studies with the TBI model, transplantation was performed immediately after lesioning, which is a relatively more straightforward way (Table 2, Figure 2G,H). Successful graft survival and integration into the host neural circuit could be achieved. Kitahara et al. demonstrated a 1-week delay versus no-delay organoid transplantation in TBI mice [25]. They noticed more remarkable graft survival, larger graft volume, and more graft axons extending along the host cortical spinal tract at 12 wpt when transplanting organoids 1 week after lesioning, compared with transplanting post-injury immediately, which is in accordance with an embryonic cerebral cortex transplantation study [75]. A possible underlying mechanism might be the secretion of pro-angiogenic factors by cells surrounding the lesion or the modulation of inflammatory responses to achieve an optimal environment for the grafts. Their following study also supported this hypothesis with transcriptomic evidence [76]. The other two TBI studies also transplanted the organoids with a 1-week window to avoid the local inflammatory storm, and the results showed good differentiation, axon projection, induced vascularization, and behavior improvement [15,29].

On the other hand, one stroke study showed the opposite results [16]. They compared transplanting hCOs 6 h, 24 h, and 7 days after middle cerebral artery occlusion (MCAO). They found hCO transplantation at 6 h and 24 h after MCAO significantly reduced infarct volume and improved neurological function and beam walking performance. However, transplanting 7 days post-MCAO had no such observed benefit. They proposed that a glial scar formed surrounding the transplantation site 7 days after MCAO might hinder the graft survival and migration. In contrast, two other stroke studies that performed organoid transplantation 1 week after photothrombotic stroke achieved positive results: grafted hCOs survived and differentiated well, integrated into the host neural circuit, and restored sensorimotor function [17,31]. Differences between these two stroke studies and the one against 7 day-delay transplantation were the stroke models and the transplantation procedures. The former two directly injected the organoids into the junction of the infarct core and the peri-infarct zone without making additional cavities. Those might lead to different results, including the effects of the glial scar. Further studies are needed to explore the mechanisms.

### 7.3. Procedure

A standard stereotaxic frame setup is required to fix the head and deliver implants precisely. Proper general anesthesia is necessary, and inhaled isoflurane is the most common choice for rodents. The intraperitoneal injection of tribromoethanol has also been used [22]. Ketamine and xylazine via intramuscular administration could be administered for the primates’ anesthesia [25]. Because of the size issue of organoids, as aforementioned, sometimes they are challenging to be injected by needle-like implantation. After a small craniectomy or craniotomy and the careful removal of the dura, most of these studies needed to make a cavity at the target region of the brain (Table 4). This can be performed by aspiration, biopsy punch, or using surgical impactor tips to create a 1–3 mm diameter and 1–2 mm depth cavity in the cortex. Hemostasis is crucial in this step, and potential brain tissue edema should be monitored [68]. The cavity size should depend on the organoids, and the depth of the cortex could extend deep to the corpus callosum. Too big of a crack in the subcutaneous connective tissue might lead to implant failure because the grafted organoids would be pushed out by cerebrospinal fluid outflow [15]. Overall, the brains would sustain some degree of traumatic injury, even though some of the studies were not designed to induce any brain damage. Several studies directly injected organoids with a Hamilton syringe using a 22–23-gauge needle or a glass capillary without making a cavity beforehand [19,26,33,37]. This procedure is preferable when transplanting into deep brain regions. After organoid transplantation, only one study used low-melting agarose and adhesive glue to immobilize the organoid grafts in situ [22], while others did not perform direct fixation of the grafts. Some of the studies covered the craniectomy lesion with the original skull bone flap or piece and sealed with bone wax. Custom head plates made of polydimethylsiloxane [20] or titanium [34] have also been used with bone or dental cement fixation. Three studies covered the skull defect with a coverslip to easily observe and measure the grafts, making in vivo two-photon microscopy feasible [12,34,35]. Some other studies with direct injection methods did not mention how they managed the skull lesion, probably with no cover, since the holes on the skull were tiny [12,26].

Appropriate postoperative care was recommended to improve animal welfare and outcomes. Some studies even gave analgesics, such as nonsteroidal anti-inflammatory drugs, for better pain control [12,21,68]. Postoperative systemic antibiotics were rarely used, even in immunodeficient rodents, since it is a “clean” surgery [77]. Only one study administered penicillin and streptomycin to SCID mice after the operation [22]. Some other studies applied erythromycin ointment locally to prevent surgical wound infection [14,16]. Of note, preoperative prophylactic antibiotics have in some cases been used in primate [25] or some rodent surgeries [27]. To our knowledge, no study has compared the outcomes and influences of using these drugs or not in brain organoid transplantation. Additionally, immunosuppressants were administered to the immunocompetent animals days before transplantation and during the whole postoperative period. Cyclosporine A for rats and Tacrolimus for monkeys were reported in organoid transplantation studies [14,16,20,25].

For more details on the transplantation procedures, some protocols that have been published recently could be helpful references [68,70].

## 8. Outcomes

What are the outcomes after brain organoid transplantation? Here we have summarized the current findings with this technique (Table 5). For practical information on some outcome measurements, there is a useful protocol published recently, including magnetic resonance imaging (MRI), immunohistochemistry, single-nucleus RNA sequencing (snRNA-seq), electrophysiology, two-photon calcium imaging, and optogenetic behavioral assay [70].

### 8.1. General Survival

The overall host animal survival rate was reported to be around 80% at 2 mpt in TBI models [15,20] and more than 90% in non-injured and stroke studies over 5 mpt [12,17].

Graft survival was assessed with immunochemistry. All studies involved xenotransplantation—human to animal. Implants were distinguished with human markers, such as human nuclei (hN), STEM 121 (human neurite marker), STEM 123 (human astrocyte marker), and hNCAM (human neural cell adhesion molecule). Co-labeling the organoids with green fluorescent protein (GFP) by lentivirus infection or directly using a GFP+ stem cell line has also been a standard method to make grafts more visible. Overall, the organoid grafts could survive in the host brain for up to 8 mpt [19], and the survival rate could range from 80% to up to more than 90% in rodent models [17,19,20]. The graft survival rate in primate models may be lower. One study reported that no graft survived in two out of four cynomolgus monkeys [25]. Apoptotic cells were much fewer in the grafted organoids than in vitro ones, which may be caused by phagocytic clearing in vivo, reduced metabolic stress, and better oxygenation and nutrition support by vascularization, which we discuss later [12,13,33].

The MRI is a useful tool for monitoring engraftment in vivo. High intensity can be seen on T2-weighted images to identify the grafts, making it possible to examine the graft position and trace its dynamic growth in living animals [15,19,25].

### 8.2. Cell Differentiation and Maturation

Nearly all studies stained the engraftment to check the cell composition, maturation, and differentiation post-transplantation. For example, Mansour et al. showed that grafted hCOs (40–50 DIV old when transplanted) expressed progenitor markers SOX2 and mature neuron marker NeuN at 14 days post-transplantation (dpt) and could last 8 months. NeuN+ cells with other mature neuron markers SMI312 and MAP2 became prominent at 50 dpt and 90 dpt. They also checked mature astrocyte marker GFAP, S100β, and oligodendrocyte marker OLIG2, revealing a noticeable amount of GFAP+ cells after 50 dpt and OLIG2+ cells after 90 dpt. However, no myelination marker, myelin basic protein (MBP), was detected [12]. Cao et al. transplanted 50 DIV hCOs into mouse brains and revealed progenitor markers PAX6 and SOX2 on 45 dpt. On 60 dpt, NeuN, deeper layers markers TBR1, FOXP2, and CTIP2, and upper layers markers SATB2 and BRN2 could be observed, with a few cells expressing human STEM123 and GABA in the grafts. On 80 dpt, STEM 121 and synaptophysin were positive [17]. Dong et al. examined their grafted hCOs and revealed the expression of SOX2, FOXG1, STEM121, hN, hNCAM, NEUN, and TUJ1 around 1 to 2 mpt. At 3 mpt, the expression of TBR1, FOXP2, SATB2, BRN2, and glutamate (GLU) was observed, indicating maturing cortical excitatory features. Around 5% of grafted cells were positive for GFAP. They detected a small proportion of the grafted cells expressing MBP, indicating myelination. In addition, NANOG+ (pluripotent stem cell marker) and Nestin+ (NSC marker) cells were nearly absent at 1 and 3 mpt, respectively [26].

In Cao’s study, they further compared 50 DIV hCOs on 45 dpt with organoids cultured in vitro for 95 days. They noticed that the percentage of cells with progenitor markers remained significantly higher in the in vivo condition, suggesting that the maturation of organoids slowed down in the host brain [17]. In contrast, Mansour et al. suggested that the in vivo environment enhanced the cellular maturation of organoids based on higher numbers of NeuN+ cells in the transplanted hCOs at 50 dpt compared to the age-matched in vitro ones. They also noticed no significant differences at earlier stages [12]. The main difference between these two studies is that the former transplanted to stroke mouse brains with a 1 delay, while the latter was implanted into normal mouse brains immediately after aspiration lesioning, which might be the reason for these contradictory findings. More studies to explore the mechanism are needed. Facilitated differentiation was also supported by the finding that GFAP+ astrocytes and Olig2+ oligodendrocytes existed in the transplanted organoids at 2 and 4 wpt, while they were still scant or absent in the age-matched in vitro ones [13].

One study transplanted 7 DIV premature hCOs into the striatum. After 2 months, MAP2+ mature neurons and GFAP+ astrocytes could be detected, and ventricular zone (VZ)-like structures could be seen, which were neither observed in the dissociated cell transplantation group nor mentioned in other studies. Graft-origin mature choroid plexus cells and PDGFRβ+ pericytes were also found but not observed in the in vitro organoids. They conducted snRNA-seq and, surprisingly, revealed that the grafted organoids expressed striatal markers ZBTB20 and STRN3 instead of the cortical markers seen in the in vitro organoids [33]. Xu’s study also supported these findings. They demonstrated that the grafted organoid cells would be affected by host niche factor regulation and align the fates with adjacent brain regions by comparing the single-cell transcriptome of the brain organoids implanted into the PFC and hippocampus at 2 and 4 mpt. The graft-origin neurons and astrocytes were more mature in the PFC than in the hippocampus, which was probably caused by extensive dopaminergic and cholinergic signaling. They also noticed that the main developmental stage of grafted human astrocytes was 4 mpt in their study [37].

Compared to hCOs, the study using hDFOs discovered plentiful PAX6+ and fewer SOX2+ neural progenitors in the grafts at 2 mpt. The percentage of PAX6+ neural progenitors decreased over time, whereas that of mature NeurN+ neurons increased from 1 to 3 mpt. The cortical layer markers CUX1, SATB2 for II/III, Necab1 for IV, and CTIP2, TBR1 for V/VII could be identified in the organoid grafts at 1 mpt. The number of CUX1+, SATB2+, and CTIP2+ cells increased over time [20]. However, the distinct layer architecture of the organoid, which appeared in vitro, was not observed in the grafts, which was also noted in Mansour’s and Daviaud’s studies. For longer-term follow-up in vivo, Revah et al. disclosed NeuN, a cortical progenitor marker PPP1R17, glial lineage cell markers SOX9 and GFAP, and oligodendrocyte progenitor cell marker PDGFRα in HN+ cells at 8 months of differentiation. Host ECs and microglia were found throughout the graft. Their findings also supported the loss of anatomical lamination architecture despite the presence of cortical layer markers SATB2 and CTIP2. They further analyzed the snRNA-seq of stage-matched hDFOs. Compared to in vitro ones, transplanted hDFOs lacked GABAergic neurons, with the presence of oligodendrocytes at around 8 months of differentiation. In addition, the cortical glutamatergic neurons of transplanted hDFOs showed advanced transcriptional maturation. They also compared the morphological differences between in vitro and in vivo hDFOs. The transplanted organoid neurons were much larger, with more dendrites, higher dendritic spine density, and longer total dendrite length than organoids in vitro [19].

Regarding the hMOs in vivo, Zheng et al. revealed mDA-specific markers FOXA2, NURR1, and TH, and the A9 DA neuron marker GIRK2 at 6 wpt. The proportion of engrafted cells co-expressing TH increased from 2.57% to 14.11% during 6 and 12 wpt. Also, 90% of TH+ cells were co-labeled with GIRK2, which suggested that A9 midbrain DA neurons had survived and matured. No graft-derived astrocytes, oligodendrocytes, or other types of neurons, such as glutamatergic neurons, were seen at this stage.

### 8.3. Axonal Projection, Cell Migration, and Synaptic Formation

Some studies have noted axonal projections and cell migration from the grafted organoids. This could be observed by using human-specific markers, such as hNCAM, or GFP labeling. After hCO or hDFO transplantation to the rodent cortex for 3–6 months, graft-derived axons could be seen in the ipsilateral striatum, internal capsule (IC), cerebral peduncle (CP), brain stem, and as far as the contralateral cervical spinal cord (SC) along the host corticospinal tract. The grafted organoids could also extend fibers bilaterally to the corpus callosum (CC), hippocampus, and contralateral striatum [12,17,25]. Moreover, axonal projections were found in the ipsilateral sensory cortex (S2) and thalamus, such as the ventral posterior nucleus (VPN), spinal trigeminal nucleus (a somatosensory nucleus) [17], and contralateral cortex [12]. In addition, Jgamadze et al. discovered minimal MBP around the projections, suggesting the lack of myelination at 2 mpt [20], which was compatible with Mansour’s findings, as previously described. Li et al. demonstrated that both LIUS and ES pretreatment could enhance axonal projections of organoid grafts with more and longer fibers, and earlier axonal outgrowth than non-pretreated control groups [28,38].

Cell migration from the graft could be found bilaterally in the cortex, thalamus, hippocampus, and subventricular zone along the CC during a follow-up period of 1–2 mpt [14,16]. Conversely, Jgamadze et al. found that mostly astrocytes and oligodendrocytes in the graft had migrated to the host, but no host neurons migrating into the organoid grafts were noticed at 2 mpt [20]. When applied to the nonhuman primate model, graft-derived axons could reach ipsilateral CC and striatum, but not more distal parts, such as the IC or CP, at 12 wpt [25]. Moreover, several studies provided evidence of synaptic connections between graft and host, showing presynaptic marker SYN and postsynaptic marker postsynaptic density protein 95 (PSD-95) [12,14,16,17,20,26].

Jgamadze et al. [20] transplanted hDFOs to the visual cortex and used virus-mediated trans-synaptic tracing methods to confirm synaptic connectivity. The modified rabies virus (RABV) system for monosynaptic retrograde tracing and the modified herpes simplex virus (HSV) system for polysynaptic anterograde tracing were applied. They found that the graft in the visual cortex received afferent connections from the adjacent cortex, ipsilateral hemisphere, bilateral hippocampus, and thalamus, especially the lateral geniculate nucleus, and was not limited to the structures of the visual network. The evidence of the efferent connections was seen in the visual cortex adjacent to the graft, ipsilateral lateral geniculate nucleus, and contralateral optic nerve [20].

After hMO transplantation to the striatum, graft axons extending from the striatum to the cerebral cortex along the CC, the globus pallidus, VP, and medial forebrain bundle, and eventually as far as the posterior cerebral peduncle, were found at 6 weeks post-transplantation. These fibers could cover the claustrum and endopiriform nucleus, even reaching the rostral olfactory bulb and the substantial innominata, and several fibers could extend along the corpus callosum into the contralateral cortex at 12-week follow-up [32].

### 8.4. Electrophysiological Neuronal Activity and Functional Integration

In addition to anatomical connectivity, functional integration is important. One crucial function of neurons is electrophysiologic activity, which can be assessed using the whole-cell patch–clamp technique or multielectrode arrays. Electrophysiological characteristics of the engrafted organoids have been assessed. Bao et al. recorded spontaneous potentials and action potentials (APs) under stimulation at 70 dpt [15]. Dong et al. also disclosed that the neurons of the grafted hCOs presented APs in response to membrane depolarization at 3 mpt [26]. The AP threshold and resting membrane potential became more negative, accompanied by a decrease in input resistance and an increase in AP amplitude at 5 mpt, indicating the progressively mature feature in a time-dependent manner. Mansour et al. also revealed the advanced electrophysiological maturity of 115 dpt grafts compared to 50 dpt ones, consistent with the temporal progressive neuronal maturation [12]. Cao et al. achieved similar results, stating that grafted hCO neurons exhibit electrophysiological properties of mature neurons on day 180 post-transplantation [17]. Revah et al. further compared the electrophysiological properties of transplanted hDFO neurons with those of in vitro ones. They found that the transplanted ones had higher membrane capacitance, more hyperpolarized resting membrane potential (by approximately 20 mV), higher maximal firing rates elicited by current injections, and higher spontaneous excitatory postsynaptic current events (EPSCs) at 8 months of differentiation. These properties were consistent with the aforementioned morphological features, suggesting that the increase in functional excitatory synapses is associated with the increase in the dendritic spine density of the graft neurons [19]. The grafted hMO neurons also showed electrophysiological properties with regular spontaneous APs with a long duration of single AP activity (>10 ms) 6 weeks after transplantation [32].

With in vitro LIUS pretreatment, the grafted organoids had higher electrophysiological activity maintained from 3 to 5 mpt [38]. Similarly, the ES pretreated organoids were also shown to have more mature electrophysiological features over time, with higher entropy of local field potentials (LFPs) than the control group, indicating an enhancement of electrophysiological maturation and complexity [28,36]. Wilson et al. discovered that transplanted organoids had a greater reduction in spontaneous neuronal activity with anesthesia than that of the surrounding host cortex at 3 wpt, and the organoid activity was generally lower in the awake state as well. These results suggested that the grafts could receive local innervation but lacked input from long-range projections at this timepoint [27,28].

Two-photon calcium imaging has also been used to assess neuronal activity. Pretreated organoids expressing a calcium indicator by viral vector infection were implanted. The results showed that grafted organoid cells exhibited synchronous, rhythmic neural activity from 78 to 150 dpt with increasing relative changes in fluorescence over time [12,19]. The calcium imaging also revealed higher neuronal activity with a synchronized pattern in ES-pretreated organoid grafts [28].

Optogenetic stimulation has been utilized to investigate the functional connectivity in further detail. One of the standard settings is to make organoids for transplantation expressing channel-rhodopsins 2 (ChR2) with fluorescent protein by viral infection. Then, pulsatile light with a specific wavelength (blue light of 470 nm for ChR2) was used to activate the grafted organoid neurons, and recordings were made from the host postsynaptic neurons to distinguish the synaptic efferents from the organoid neurons to the host neurons. Several studies have successfully recorded postsynaptic currents by stimulating the hCO engraftment at 5 to 6 mpt [12,17,26]. Conversely, one could make host neurons expressing the ChR2-fluorescent protein, optogenetically stimulate them, and record from the graft to detect afferent connections. Cao’s study showed inward currents in grafted neurons in this way [17]. Taken together, it seems well-established that brain organoids can functionally integrate into the host brain neural network with afferent and efferent connections.

### 8.5. Vascularization

Another focus is the vascularization of the organoid engraftment, since the brain organoids do not have their own ECs to build up vascular systems. The grafts were found to receive newly formed vessels from the host and even promoted angiogenesis, which was observed by CD31 staining in many studies. The CD31+ vascularization was observed as early as 14 dpt, but not at 5 dpt, indicating that the organoid vascularization occurred between these time frames [12]. Increased microvessel density with positive α-smooth muscle actin staining could be seen in the lesion periphery and the grafts at 7 dpt [29]. These functional vasculature networks could reduce apoptosis in grafts and improve the graft’s survival compared to in vitro ones, as evidenced by the observation that these benefits occurred after vascularization [12]. Organoids pre-modulated by LIUS or ES were found to have more vascular infiltration from the host after transplantation [28,38]. Some studies even introduced ECs into the organoid culture to improve vascularization. Both iPSC-derived ECs and human umbilical vein EC-vascularized organoids could give rise to graft-derived vascular connections with the host, which could be seen from 14 dpt, and improved graft survival compared to non-vascularized ones [21,22]. Shi et al. used scRNA-seq to compare the transcriptomes of vascularized and non-vascularized organoids at 60 and 100 DIV. The analysis revealed no remarkable cell type differences between these two types of organoids. They further measured cell death by cleaved CASPASE 3 staining and found that transplanted vascularized organoids had the least cell death, followed by the cultured age-matched vascularized organoids, then non-vascularized organoid grafts at 60 dpt. Compared to the angiogenesis from the host, the graft’s angiogenesis came earlier into the graft core at 30 dpt. In addition, the vascularized hCOs were found to have larger outward current amplitudes, lower resting membrane potential, and greater cell capacitance in vitro than the control at 80 DIV, suggesting that the vascular system may have facilitated the electrophysiological functional development of individual neurons [22].

### 8.6. Host Brain Aspects—Neurogenesis and Immune Modulation

Organoid implantation can decrease apoptosis and enhance neural survival in the transplantation periphery of the host cortex. In a stroke study, this was observed by a decreased number of TUNEL+ cells and an increased number of Nissl+ neurons in the grafted rat brain at 7 and 28 dpt [16]. Moreover, organoid engraftment can be enhanced in the host. Miao’s group found increased NESTIN+ proliferated neural stem cells, DCX+ migrated newborn neurons, and NeuN+ mature neurons at 7 and 28 dpt in the ipsilateral hemisphere cortex outside the transplantation territory as well as bilaterally in the hippocampal subgranular zone (SGZ) and subventricular zone (SVZ), which are well-known areas of neurogenesis. They revealed that these newly generated neurons were predominantly from the graft (with human marker) in the ipsilateral cortex and the host in SGZ and SVZ, suggesting exogenous and endogenous neurogenesis [16]. Similar findings were observed in another study that transplanted hCOs into the retrosplenial cortex. A reduction in Fluoro-Jade B+ apoptotic cells in the CA1 and the dentate gyrus of the hippocampus and ipsilateral cortex and an increasing number of GFAP+ astrocytes around SVZ and DCX+ newborn neurons in the dentate gyrus were found at 7 and 14 dpt [29]. Additionally, Miao’s group discovered that 55 DIV hCOs had more pronounced effects on neurogenesis than 80 DIV ones. They also examined hippocampal protein expression and found an increase in the neurotrophic factors brain-derived neurotrophic factor (BDNF), nerve growth factor (NGF), and epidermal growth factor (EGF) at 14 dpt in the transplantation group, which could be one mechanism underlying the enhanced neurogenesis [14].

Inflammation-modulating effects by the grafted organoids were equivocal in disease models. In the MCAO study transplanted with hCOs, neuroinflammation indicators, including proinflammatory cytokines tumor necrosis factor α (TNF-α) and interleukin (IL)-1 β, macrophage marker CD68, and infiltrated neutrophils MPO-1 did not differ between the transplantation and stroke groups [16]. The density of Iba1+ microglia was also not different between the transplanted and control groups as noted in a TBI study [29]. No exacerbated neuroinflammation after transplantation might suggest good compatibility of grafts in the host brain without rejection. Interestingly, even the downregulation of the mRNA expressions of the proinflammatory cytokines, IL-1β and IL-6, and the upregulation of Nrf2 and Hmox1 against inflammasome activation were noted in the hMO-grafted animals in a Parkinson’s disease study [32].

### 8.7. Host Individual Aspects—Behavioral Function

Neurological function has been evaluated to further investigate the functional integration from the cellular level to the gross individual level.

In TBI studies, Wang Z. et al. transplanted hCOs to the lesioned motor cortex and showed modified neurological severity scores (mNSSs), a standard neurological evaluation for rodent central nervous injury covering motor, sensory, and reflex, and balance function [78,79]. Scores improved from 5 dpt in the transplantation group as compared to the TBI group and returned to a normal level from 21 dpt. The beam walking test, which evaluates motor coordination [80,81], was also performed better in the transplantation group during 42 days of follow-up [14]. Another study transplanted the organoids to the injured parietal cortex and conducted a Morris water maze test and a passive avoidance assay. The former assesses spatial learning, and the latter is for avoidance learning. Both are related to memory function [79,81]. The transplanted mice demonstrated better spatial learning abilities and shorter latencies and distances to the platform at 61 to 70 dpt. The swimming speed was no different from that of the control group. In the passive avoidance assay around 2 mpt, the transplanted mice preferred staying longer in the lightbox than the TBI control ones, suggesting better memory to avoid electric shock in the dark side [15]. The cognitive restoration associated with learning and memory [82] was also noted by the novel object recognition test. In this case, hCOs were implanted into the lesioned retrosplenial cortex. The preference index was significantly higher in the transplanted animals than control ones at 7 and 14 dpt, with a greater improvement over time [29]. In contrast, another study using the Barnes maze to test spatial learning and memory abilities showed no significant difference between mice grafted in the lesioned retrosplenial cortex and ungrafted mice [12]. The study in which ES-pretreated organoids were transplanted to the mouse S1 cortex conducted behavior tests, including the open field test, which assesses locomotor ability and exploration behavior [78,81], and novel object recognition tests. Another study with the same transplantation site also showed no significant behavioral difference between ES or non-ES organoids transplanted and non-transplanted groups at 3 mpt [28].

In stroke studies, better mNSSs and beam walking performance were noted from 2 to 5 dpt, respectively, in the MCAO rats that received transplantation within 24 h after stroke [16]. Another series of photothrombotic stroke studies, one of which transplanted hCOs and another hMGEOs, used a battery of tests, including the cylinder test, grid-walking test, and adhesive removal test, to assess spontaneous symmetry forelimb use, motor coordination, and sensorimotor function. Both studies achieved similar results. The transplanted group had a significantly reduced asymmetry index in the cylinder test and fewer foot faults in the grid-walking test at 150 dpt. The grafted mice also showed shorter touch and removal latency in the adhesive removal test than the stroke control ones. The above parameters of the grafted animals are even closer to those of sham animals without a significant difference at 150 dpt [17,31]. They further validated whether the grafted organoids contributed to the sensorimotor improvement by using the hM4Di/CNO model. They applied clozapine-N-oxide (CNO), a designer drug (DREADD) agonist, to silence the activity of organoid grafts with hM4D(Gi). The behavioral improvement in the three tests was partially eliminated with CNO, except for the touch latency in the adhesive removal test, indicating that grafted organoids’ neuronal activity is crucial for the restoration of sensorimotor function [17]. Of note, spontaneous behavior recovery should be considered in rodent brain injury studies [79].

In addition to the TBI and stroke models, some other studies without brain lesioning before transplantation also included behavior tests. Dong et al. injected organoids into the medial PFC, which is related to higher cognitive functions, emotions, and goal-directed behaviors, and followed open field and fear conditioning tests to assess locomotor function and associative learning and memory [83]. At 6 wpt, there was no difference between engrafted and control mice in the open field tests, including total distance traveled, the proportion of time spent in the center, and the number of crossings, suggesting that the organoid grafts did not impair the normal physiological locomotor function. In contrast, the percentage of freezing in the fear conditioning test was higher in the grafted group at 8 wpt, which meant that the startle fear response could be potentiated by organoid transplantation [26]. Revah et al. injected organoids into the S1 cortex and also found no difference between the transplanted and control groups in the open field test on 90-dpt, as well as the novel object recognition test [82]. They even showed no differences in the fear conditioning test, which is at variance with the previous study. The discrepancy could be explained by the different transplantation sites in these two studies. Further, they conducted an optogenetic behavioral assay at 90 dpt. Here, they transplanted hChR2–EYFP hDFOs and stimulated the grafts with blue (473 nm) or red (635 nm) laser through a pre-implanted optical fiber. Meanwhile, when licking behavior was conditioned with a water reward during blue light stimulation, after 15 days of training, hChR2–EYFP organoid-implanted mice increased licking during blue light stimulation compared with red light stimulation. The changes in the licking behavior were not observed in the control organoid transplantation group. These phenomena suggested that the activation of engrafted organoid cells could trigger rat neurons to drive reward-seeking behaviors [19].

Sensory input from the host to the graft has also been assessed. In the same study by Revah et al., they recorded increased activity in a subset of grafted organoid cells in response to contralateral whisker deflection [19]. Jgamadze et al. transplanted to the visual cortex and also detected visually evoked neural activity in the grafted organoid after 2 mpt. The pattern of evoked unit activity was qualitatively similar to that in the naïve rat visual cortex. However, some differences were detected between the graft and the visual cortex in response to visual stimulation. The response of grafted organoid neurons was more prolonged with several activation peaks, which may result from the increased internal connectivity of the organoids. Smaller amplitude in event-related potentials and fewer neurons exhibiting evoked activity were also noted compared to naïve visual cortex neurons [20]. Another study validated the graft response to visual stimulation. In this case, 49 to 63 DIV hDFOs with microelectrode arrays were co-implanted into the left retrosplenial cortex of mice. LFPs emerged around 3 wpt and increased over time [27]. In addition, the previously mentioned LIUS study used pain response to investigate the circuitry integration. They detected the Gamma oscillation activity from the grafted organoids while withdrawing in response to von Frey stimulation and found that the amplitude of the oscillation was significantly enhanced in the LIUS-pretreated group at 3 and 5 mpt, hence proving that the functional integration could be improved by LIUS pretreatment [38]. Similar outcomes showing pain sensation facilitation were also observed in another study in which ES-pretreated organoids were transplanted [28].

In the hMO transplantation study, the organoids were implanted into the striatum in a 6-OHDA Parkinson’s disease model, and apomorphine (APO)-induced rotation, rotarod, and open field tests were performed. In addition to the previously described open field tests, the former two assessed asymmetry in motor and posture control, as well as motor coordination and balance, respectively [81,84,85]. The results of all three tests were significantly improved in the transplantation group, suggesting that hMO transplantation can restore motor functions impaired by 6-OHDA lesioning [32].

Overall, the findings of these behavioral tests revealed that the organoid grafts could functionally integrate into host brain circuits and facilitate the certain behavioral functions specifically related to the transplantation region. Thus, carefully selecting the test batteries that correspond to the functions of the target regions is crucial for obtaining representative readouts, which rely on a better understanding of functional brain–behavior correlation.

## 9. Post-Transplantation Modulation In Vivo

Several studies have investigated the effects of in vivo modulation on organoid grafts. In the LIUS study, stimulation was applied to the grafted organoids at S1 cortex with LIUS in vivo for 1 month since 7 dpt. In addition to the beneficial effects as in vitro pretreated organoids, such as enhanced neural development, vascularization, electrophysiological maturation, and functional integration as mentioned before, the in vivo LIUS group was found to have more axonal projections in the ipsilateral host brain with wider and more uniform distribution compared to the in vitro LIUS-pretreated group. Earlier axonal outgrowth towards deep brain areas than the control group at 2 mpt was also shown [38]. The OBCI study transplanted 40 DIV hDFOs into the mouse S1 cortex and built the OBCI system at 25 dpt with one electrode inserted in the engraftment and another in the M1 cortex. Ten days of 50 μA stimulation were administered from 30 or 60 dpt. Similarly to LIUS, with 50 Hz OBCI modulation at an early stage in vivo, the organoid engraftment had a larger volume, more proliferation and synaptic formation, a higher expression of CTIP2 and SATB2, and electrophysiological maturation within 180 days of follow-up. By regulating with 70 Hz stimulation via OBCIs at a late stage, when the connection between the grafts and host tissue had been formed, more and longer axonal projections to the bilateral corpus callosum and ipsilateral hemisphere and an increase in synaptic connections from the grafts to the host were seen at 120 and 150 dpt. Electrophysiological activity, connectivity, and the coupling of LFPs were also promoted. Like in their previous modality pretreatment studies, the Gamma activity of the grafts’ response to pain stimulation was enhanced and could reach the level of the naïve group at 180 dpt, indicating functional integration and recovery. There were no deficits in memory or locomotion assessed by open field and novel object recognition tests in this case [30].

## 10. Current Applications

Brain organoid transplantation was first used in proof-of-principle studies [12,13,19,20,25,26,27,28] to see how grafted organoids react in the host brain and then widely studied for potential therapeutic usage in TBI [14,15,29,30], stroke [16,17,31], and Parkinson’s disease [32] models (Figure 2). Generally, graft survival, differentiation and maturation, functional integration, and behavioral improvement were observed in these studies, which showed this technique’s feasibility and may have therapeutic potential. Several in vitro or in vivo modalities have been shown to facilitate these effects [28,30,38]. Vascularization research [21,22], which introduced ECs, has successfully demonstrated vascular connections with the host.

To study cell interactions between graft and host, organoids were implanted in different brain regions, which revealed that grafted cell fate changes depending on the adjacent environment [33,37]. Microglia- or astrocyte-enriched organoids have been transplanted to mimic the human brain environment and study human cell interactions in vivo. The graft-derived human microglia and astrocytes within the grafts could mature and respond to inflammatory stimulation functionally [34,35]. These results proved this to be a practical platform for studying cell interactions in vivo.

Furthermore, it has been used for disease modeling and testing therapeutic approaches. The microglia-enriched study mentioned above also transplanted organoids derived from the cells of patients with autism and macrocephaly. They cocultured erythromyeloid progenitors (EMPs), the precursor of microglia, with forebrain organoids to obtain microglia-enriched organoids. They discovered an increase in the number of human microglia (hMGs) with a larger soma size and more filopodia emerging from the processes; those were associated with a reactive microglia phenotype. They further validated this effect with control hMG-enriched autism organoids and showed similar morphological changes in control hMGs, indicating that these changes were driven by the pathological brain environment instead of intrinsic microglia genetic predisposition [34].

Also, hDFOs generated from patients with Timothy syndrome (TS), a severe genetic disease caused by a mutation in the L-type voltage-sensitive calcium channel CaV1.2, have been transplanted. Abnormal dendritic morphology was noticed in vivo after TS DFO engraftment, associated with increased synaptic spine density and a higher frequency of spontaneous EPSCs [19]. These grafted human TS DFOs in the rat brain have also been used as a platform to test antisense oligonucleotide (ASO) therapy. Chen et al. reported that the expression of CACNA1C exon 8A, which with the heterozygous c.1216G>A pathogenic variant is the cause of TS type I, in the transplanted TS organoids would reduce after 14 days after intraventricular injection of antisense oligonucleotide (ASO.14). The cellular dysfunction with increased post-depolarization residual calcium of TS neurons could also be restored, and the dendrite morphology defects could be corrected [36]. Another study transplanted abnormal spindle-like microcephaly-associated protein mutant organoids, whose mutation is the most common cause of recessive microcephaly, and tried to rescue the NPC depletion by in vitro or in vivo LIUS treatment. The results showed that the graft areas, vascularization, NPC proliferation, neurogenesis, and electrophysiological activity were superior in the in vitro and in vivo LIUS-treated group as compared to the control group. A wider range of axonal projections was seen in the in vivo LIUS group [38]. Overall, these studies provided evidence that the generation of organoids is a suitable approach for disease modeling and the development of novel interventions, most importantly being an in vivo human cell-based platform.

## 11. Safety Concerns

When it comes to an intervention, safety should always be considered. The survival rate of transplanted animals is around 80% to more than 90% for months. A main concern of transplantation is the rejection of the graft. So far, studies have used either immunodeficient animals or appropriate immunosuppressants for xenotransplantation. No major rejection event was reported. Moreover, the transplantation of organoids did not lead to a major increase in neuroinflammation as compared to non-implanted but stroke-subjected animals. This, in turn, might be due to the already high level of neuroinflammation induced by brain damage [13,16,32].

Since the origin of organoids is PSCs, there is legitimate concern about the potential tumorigenicity. In theory, PSCs pre-differentiated into neural fate would reduce the tumorigenic capacity [86,87]. Currently, most of the cells of the brain organoids used for transplantation have been guided towards neuroectoderm fate and lost pluripotency before transplantation. No brain tumor formation has been reported in the grafted animals so far. One iPSC-derived hMO transplantation study even pretreated the organoids with QC, as mentioned before, to lower the tumorigenic risk by the removal of undifferentiated cells [32]. However, graft overgrowth was observed in Kitahara’s study after transplanting 6-week-old hDFOs but not 10-week-old ones [25]. This finding could be the result of the more immature cells and higher proliferative ability in younger organoids.

## 12. Current Ethics

Current organoid transplantation studies involve invasive procedures on animals and some of them use hESCs. The ethical concerns related to these issues should be carefully considered. We would like to emphasize the 3R principle of animal research, replacement, reduction, and refinement, to improve animal welfare [88]. The following section focuses on some unique ethical considerations of brain organoid xenotransplantation. The first major one is the potential “humanization” of host animals [89], since the human brain controls not only fundamental neurological capacities, such as reflexes and motor and sensory functions, but also consciousness, higher-level cognition, abstract thinking, and intelligence, which are key differences from animals. Brain organoids have demonstrated some similarities to the human brain, from genetic, epigenetic, and epitranscriptomic to developing features and cellular architectures that can recapitulate the human fetal brain development through the second trimester. Electrical activity has also been detected in brain organoids. Of note, there are still some distinct differences between the human brain and organoids, such as brain organoids lacking ECs and having restricted size due to diffusion limitation. Regardless of these differences, brain organoids are still sometimes considered as a “mini-brain in a dish” with many similarities. And transplanting these human mini-brains into animals’ brains would evoke the debate and unease concerning “Human–Animal Brain Chimeras” [89]. Would the transplanted animals become more “human”, for example, having human awareness, cognition, complex emotion, intelligence, or behavior? Current transplantation studies do not support this concern, including one study that involved transplants to monkeys [25]. Some of the studies indeed revealed enhanced or improved behavior in symmetry limbs usage, motor control, spatial or conditioned learning, and memory after transplantation as previously described, but those are still far from human characteristics. Most studies selected the motor or sensory cortex as the transplanted targets that would minimally affect higher brain functions. However, one study implanted hCOs into medial PFC, which is related to cognition, and the authors noticed an enhancement in the startle fear response as aforementioned, with no other obvious cognitive impairments or changes [26]. Another study transplanted brain organoids into the PFC and hippocampus, which are involved in learning and memory, but behavioral function was not assessed. During their 4-month follow-up, no bizarre behavioral change in animals was noticed [37].

Another issue, whether brain organoids have consciousness, is still under debate. So far, brain organoids cannot recapitulate the essential complex neural architecture for primitive consciousness [90]. The quantitative computational capacity also matters. Even using 2 g of human brain organoids to replace a whole rat brain, that is only 143 million total and 26.7 million cortical neurons. Compared to the human brain of 1200 g with 86 billion total and 16 billion cortical neurons, it is way too small to reach the computational capacity for self-awareness [91]. And after transplanting a few human brain organoids to larger host animals, it is hard to alter the higher-order cognitive abilities since the cell dose is too low compared to cell numbers in the host brain [92]. In the future, if the “conscious” or “sentience” (the capacity to feel pain and pleasure) brain organoids have been developed or detected, the moral status of these organoids should be granted [90,93]. The moral rights of transplanted animals need to be carefully discussed [90]. Back then, instead of focusing on “humanization”, Chen et al. suggested that we should pay attention to the possible enhancement of chimeric animals, which has been observed in some studies, and the elevation of their moral status [89,90,94]. Appropriate and tailored welfare protections should be provided for these chimera animals [89,95]. In response to these complicated issues arising from this novel technique, the National Academies of Sciences, Engineering, and Medicine of USA has released a consensus study report from the Committee on Ethical, Legal, and Regulatory Issues Associated with Neural Chimeras and Organoids to address ethical concerns and to summarize aspects related to the governance of research with human–animal chimeras, included general animal welfare, the distinctions between human beings and animals, and about the enhancement of brain functions and consciousness [96,97].

## 13. Challenges and Future Perspectives

### 13.1. Cell Diversity

One limitation of current brain organoids is, in general, the lack of most cell types derived from lineages other than neural ectoderm, such as ECs and microglia, which are essential components of the in vivo brain [4]. (Of note, one study reported innate microglia developing in the tissue with a modified non-guided protocol [98]). Hence, more and more studies have tried to enrich the cell component and even the structure of the brain organoid in several ways. For example, ECs have been introduced into the brain organoid by co-culture and successfully generated a vascularized organoid and vessel connection with the host [21,22]. Vascularized brain organoids could also be achieved by creating assembloids with brain and vessel organoids in vitro [99,100,101]. Microglia, the critical resident immune cells in the brain, have also been cocultured with brain organoids to generate microglia-containing human brain organoids [102]. Coculture with EMPs was also feasible [34]. Innate microglia derived from mesoderm-derived progenitors within hCOs were also reported, as noted previously [98]. In addition, since astrocytes and oligodendrocytes appear rather late in the standard organoid-generating protocols [4], some studies have used batches of growth factors or small molecules to accelerate astrogliogenesis [35] and oligodendrogenesis [103,104]. Furthermore, more complex models are being developed by assembloid methods [49,105].

### 13.2. Heterogeneity, Necrotic Core, and Quality Control

Another problem when it comes to transplantation for therapeutic intent is the heterogeneity of organoids. Every organoid has different cell composition, maturity, and architecture; some even have necrotic cores, which makes it much harder to standardize than drugs or stem cells, and such standardization is crucial in clinical applications. The necrotic core is mainly caused by restricted oxygen and nutrient exchange [46,60], limiting the maturation and maximal size of organoids [46,106], and becomes one of the major hindrances when translating to the clinic. Some bioreactor methods were introduced to mitigate this problem, but high shear stress may cause cell damage [46,54,107,108]. Another solution is mechanical cutting with or without an air–liquid interface platform, which can significantly reduce cell apoptosis, but it leads to destruction that would destroy the 3D integrity of organoids [65,66,109]. Fortunately, there are some novel developing technologies which are discussed later.

Currently, the general selection criteria of brain organoids for transplantation are mostly based on the gross appearance of the organoids [67]. Non-invasive methods to analyze the living organoid component for quality control are very limited. In other words, it is nearly impossible to know the exact composition of the organoid that is selected to be transplanted before grafting. In a retina organoid study, they dissected the organoid spheroid into caps and rings and analyzed the composition and gene expression to exclude off-target tissues. They found comparable results from caps and rings and suggested that we could use rings for quality control and select the corresponding caps for further retinal sheet transplantation [94]. Although sufficient inside quality control is not feasible now, one unpublished study used machine learning-based classification by 35 features of bright-field images (e.g., area, circularity, perimeter, etc.) to improve the current gross selection criteria [110]. Developing a reliable quality control method is pivotal for clinical translation in the future. Furthermore, before moving to clinical usage, good manufacturing practice (GMP)-compliant animal-free production is necessary [111]. Unlike some other types of organoids, such as the pancreas [112] or salivary gland [113], no GMP method has been available regarding the manufacturing of brain organoids so far. The recently published literature has provided some practical guidelines for the manufacturing and application of organoids, including brain organoids [114]. A GMP-compliant method of neural-related retinal organoids has been discussed [115]. In addition, some techniques to scale up brain or midbrain organoid generation have also been reported [116,117].

### 13.3. Time Issues

Because the maturation of the human nervous system takes years [118], the grafted brain organoids may not fully mature within the time frame of current studies. Longer follow-up may be needed to observe the long-term influence [20].

Since the brain organoids are young and immature, their use for modeling (not for treatment) an aging brain or neurodegenerative diseases will be a challenge [119]. Some strategies have been developed regarding this issue, such as selecting aged or patient iPSC sources, inducing cellular senescence, epigenetic modification, mitochondrial or vascular dysfunction, protein aggregation, oxidative stress, and immune response, manipulating the environment, and extending culture time, to generate aging or degenerative phenotype organoids [120,121].

Timing is another potential obstacle to clinical therapeutic application. The optimal timing of cell replacement therapy for stroke or traumatic brain injury is still controversial. The acute phase may encounter a toxic inflammatory post-traumatic environment, while the chronic phase may face glial scar and decreased neuroplasticity [122,123]. Some preclinical and clinical stem cell studies suggest that earlier treatments (within a few days) provide more significant benefits [124,125,126]. If so, organoid transplantation will miss the optimal therapeutic window due to the long culture period of organoids. Developing a faster protocol to accelerate the differentiation may be an idea [127,128], but so far, it still needs to be faster to meet the window. Another method is to cryopreserve the brain organoids, ready-to-use, to make them available when needed. However, this is a great challenge since brain organoids are quite fragile to temperature changes [114]. Interestingly, a graduate thesis showed it was possible to cryopreserve brain organoids at all differentiation stages, with younger brain organoids being preferred [129]. A more recent publication provides a promising regimen for the cryopreservation of neural organoids. The authors used the MEDY medium, which contained methylcellulose, ethylene glycol, DMSO, Y27632, and organoids maturation medium, to cryopreserve human neural organoids. They showed that it was feasible to cryopreserve the hDFOs aged 28 days to 105 days and that organoids could be preserved for 1.5 years. Ventral forebrain organoids, spinal cord organoids, and optic vesicle organoids have also been tested successfully [130]. With these practical cryopreservation methods, not only could the time issue be potentially solved but also the cost could be reduced.

### 13.4. Translational Differences

A general concern is the translational differences between animal models and human brains, e.g., differences in genetics, anatomy, physiology, pathological response, cognition level, and even behavioral performance [131]. For example, the considerable brain size difference affects the dose and response [124], the difference between animal disease models and human disease pathology, and the great degree of spontaneous recovery in many behavioral tests of animal models, which was not observed in humans [79]. Currently, all brain organoid transplantation studies have been conducted in animal models, mostly in young rodents. How can we interpret those outcomes and apply them to a broader population? More studies to explore appropriate models and behavioral tests will be necessary to overcome the translational barriers.

### 13.5. Essential Brain Injury

The next practical issue is related to the transplantation procedure. The commonly used procedures require craniectomy since the organoids are much bigger than the cells. Making a cavity in the brain has, in some cases, been needed for transplants. This artificial injury should be a serious concern as it may interfere with normal neurological functions. Alternatively, as reported by several studies, microinjection with smaller organoids is feasible to minimize the destruction of brain tissue [19,26,33,37]. Especially when transplanting into deep brain regions, this technique is recommended. Moreover, one study suggested that a cavity is not required when transplanting into neonatal rats [70].

### 13.6. Ethics for the Future

Given the numerous challenges and concerns as aforementioned, it may be too early to discuss ethical issues related to future clinical applications. Each concern could raise an ethical question, such as safety, quality, and essential brain injuries, in addition to fundamental informed consent. As in research ethics, the unique considerations of the brain organoids, whether they have consciousness or the capability to alter the recipient’s mental or higher cognitive functions and their moral status, make ethical issues even more complicated [132,133], particularly in the context of allogeneic transplantation. In this scenario, the informed consent also becomes more complex due to the involvement of the donor [133]. Now it is still far from clinical applications. Before taking this step, more substantial evidence, expert reviews, and professional discussion are needed to address ethical issues in the future.

### 13.7. Brain Organoids on Chip

Thanks to bioengineering innovations, some limitations or challenges of current organoid technology are expected to be overcome. Organ-on-a-chip (OoC), an advanced culture model, utilizes microfluidic chips to provide a more accurate microenvironment for culturing cells. The chips are made from biocompatible materials by photolithography or 3D printing technology to create a microfluidic system. Considering biocompatibility, flexibility, permeability, and transparency, polydimethylsiloxane (PDMS) is the most common material choice [134]. With this system, biochemical gradients, oxygen tension, and mechanical forces can be precisely controlled [135]. The first OoC concept was announced in 2010, which was the lung-on-a-chip [136]. Then, Lancaster et al. introduced this microfluidic chip method into brain organoid generation [137]. Since 2017, more studies have been published. Zhu et al. used a PDMS micropillar array to offer identical spaces for embryoid bodies formation, resulting in more consistent morphology and neural differentiation, but did not improve the nutrient exchange in that model [138,139]. Wang et al. developed a perfusable microfluidic system which allowed nutrients and gases to perfuse through the microchannels and facilitate exchanging via micropillars. They demonstrated larger and more continuous neuronal organization of the cerebral organoids on the chip compared to those with conventional plate culture. Moreover, the expression of TBR1 and CTIP2 was also enhanced, indicating the improvement of cortical development. Forebrain and hindbrain regionalization was also achieved [140]. Cho et al. applied another pump-free hydrogel-infused microfluidic device, which allowed the perfusion of media driven by a rocking platform to mimic dynamic microfluidic niches in the brain. Similarly, by superior controlled fluid flow, the exchange of oxygen, nutrients, and bioactive molecules, neurogenesis, and corticogenesis were enhanced. Consequently, the hCOs were able to develop more complex structures with volumetric expansion and could be maintained for up to 120 days. In addition, the electrophysiological properties were also improved compared to the plate-cultured organoid, with a higher average amplitude of Na+ currents and AP firing rate [108]. Another type of microfluidic chip contains an air–fluid interface, which allows the organoid to grow in atmospheric oxygen levels. Higher hCO viability was reported with this system [106,141]. Not only for the hCOs or hDFOs, but OoC technology has also been used in hMO culture and could enhance dopaminergic neurogenesis. Of note, here they used a millifluidic system instead of a microfluidic one [142]. Importantly, most of the studies showed the same feature: compared to conventional culture, the necrotic core was significantly reduced with the OoC system due to better nutrient perfusion and oxygenation. Furthermore, with the uniformity control by these devices, the organoid heterogeneity and batch variability could be substantially attenuated, hence improving the reproducibility [106,108,141], which could respond to the previously addressed “Heterogeneity, necrotic core, and quality control” issue.

More complicated practices can be conducted on the OoC platforms. Salmon et al. successfully generated vascularized hCOs by culturing organoids on a chip surrounded by the microchannel carrying flowing hPSC-derived pericytes and ECs [143]. Engineering assembloids from multiple brain regions were also feasible. With delicate control of the signaling gradients on the chips, forebrain organoids with multiple domains could be created with topographic patterns [144]. More advanced designs are also being developed. Three-dimensional microelectrode array (MEA) to record stereoscopic electrical properties and other biosensors to monitor optical, chemical, mechanical, and thermal information are being incorporated into the OoC system, which can provide a better understanding of the physiologies and finer control of microenvironments [135,145,146,147,148]. To the manufacturing extent, the OoC system enables automatic processes that minimize manual interference and can achieve higher throughput production [106,137]. Taken together, the OoC system could be a practical way to approach GMP-compliant manufacturing.

Current applications of brain-on-a-chip are mainly focusing on the proof-of-concept of bioengineering, brain development, disease modeling, and substance exposure platforms [106,108,138,140,141,142,143,149,150,151,152]. Furthermore, the cerebral organoid on an MEA chip has never been used as a biological neural network for artificial intelligence computing [153]. However, the research on transplantation applications is still lacking. Given the strengths of the OoC system in process optimization, it has strong potential to improve the quality of transplantation, thereby taking a step toward clinical application. In addition, more advanced organoid chips to build up OBCIs in vivo for functional study may be possible in the future. Despite these advantages, the key challenge to consider is the high technical requirements for chip design and fabrication (Table 6).

## 14. Limitation of the Review

As this field of brain organoid transplantation is newly developing, the current published studies were explored its wide potential and designed to prove the concept for various purposes. The heterogeneity among studies is considerable, including the type and the age of organoids, the site and procedure of transplantation, age, sex, species, and strain of the recipients, and outcome measurements. And the sample size was usually small. Randomization and blinding were not addressed or applied in most of the studies, and some more information for bias assessment is lacking (Table 3). Hence, the risk of bias cannot be excluded. Despite these shortages, some key common features could be reproduced in different studies, so that the brain organoid grafts could survive and functionally integrate into the animal host brain. However, in order to obtain solid evidence or conclusion, well-designed randomized controlled studies are necessary. Moreover, the current publications are all animal studies. The applicability of the results to humans is still questionable due to the big translational gap as described previously.

## 15. Conclusions

Many effects of brain organoid transplantation are now well-documented: good graft survival, promising neuronal differentiation and maturation, filling the tissue defect, axonal projection to adjacent host brain and distant brain regions, even along the corticospinal tract, proper vascularization, inducing neurogenesis, and functional integration into the host neural circuit with functional electrophysiological and synaptic connection, both afferent and efferent, improving or potentiating some behaviors, such as sensory, motor, learning and memory.

Both hiPSC- and hESC-derived organoids can be used for transplantation. Transplanting 7–90 DIV cerebral or forebrain organoids or 15 DIV hMOs is feasible. Modified organoids with GFP or ChR2 expression can also be used. Organoids enriched with cells other than neuroectoderm have also been transplanted. To note, younger organoids may result in graft overgrowth, while older ones have lower cell survival and neurogenesis [14,25]. The choice of selecting an organoid generation and differentiation protocol should be based on the objectives and goals of the given study [4]. Some additional pretreatments of organoids to prevent infection, tumor formation, and cell death and facilitate organoid development are available. Transplanting to young adult or neonatal rodents with or without immunodeficiency and even monkeys is possible. In brain injury or stroke models, the best timing for transplantation still needs to be determined. A few hours to one week after injury may be appropriate. The disease mechanisms and pathophysiological microenvironment changes over time should be considered. The target sites for transplantation should be based on the interests of the study, which are related to specific neurological functions. The transplanting procedure requires craniectomy or craniotomy and could be performed either by creating a cavity or microinjection, depending on the size of the grafts. A proper cover for the skull defect may be needed. Commonly used outcome measurements could include but are not limited to immunochemistry staining for human cells to see graft survival and the specific markers of stem cells, neural progenitors, mature neurons, mature or immature astrocytes, oligodendrocytes and myelin, cortical layer markers, synapse, ECs, etc., and snRNA-seq to assess differentiation, maturation, and axonal projection; virus synaptic tracing to prove synaptic formation; two-photon calcium imaging to evaluate neuronal activity; electrophysiological studies with or without optogenetics to measure the functional electrophysiological integration; and corresponding behavior tests to assess the effects of neurological function.

Although brain organoid technologies are emerging as promising strategies for treating neurological disorders, they differ significantly from cell transplantation in their current stage of development and clinical applicability. Cell transplantation-based approaches using stem cells, reprogrammed cells, or induced neural progenitors have advanced considerably and are already undergoing preclinical and clinical evaluation. For example, dopaminergic neurons derived from embryonic or induced pluripotent stem cells are currently in clinical trials for the treatment of patients with Parkinson’s disease [154,155]. These therapies offer well-defined protocols for differentiation, delivery, and safety, including tumorigenicity screening and targeted, minimally invasive stereotactic implantation. Transplanted cells, such as neural stem/progenitor cells or induced neurons, have been shown to integrate into injured brain circuits, form synaptic connections, and contribute to functional recovery and regeneration. Moreover, cell therapies can be tailored to replace specific neuronal or glial populations, such as excitatory projection neurons, inhibitory interneurons, or oligodendrocytes, thereby addressing the heterogeneous cellular loss often seen in neurodegenerative and traumatic brain conditions.

**Figure 3:**
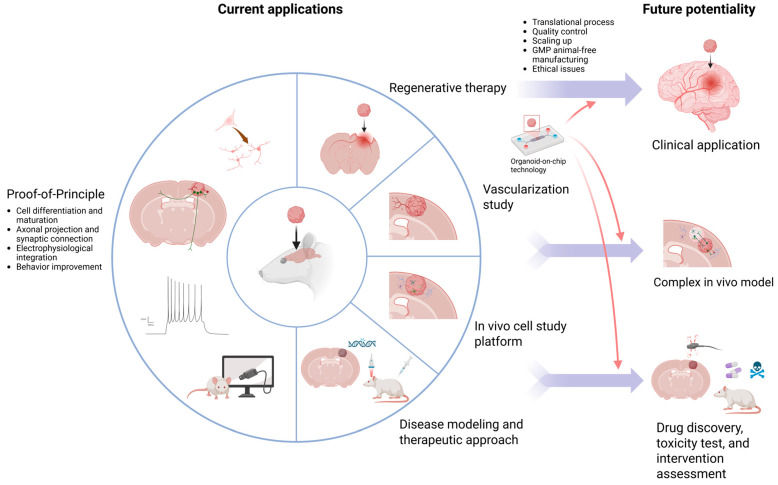
Current applications and future potentials of brain organoid transplantation.

Brain organoids, while offering exciting future possibilities, are currently better suited for disease modeling, drug screening, and investigating human-specific neurodevelopmental processes (Figure 3). They have been applied in various preclinical studies, including models of traumatic brain injury, stroke, Parkinson’s disease, and spinal cord injury [19,34,35,37,38]. Some studies suggest that organoid transplantation may support better graft survival, angiogenesis, neural proliferation, and axonal growth as compared to cell suspension [156]. However, these claims remain largely unsubstantiated due to a lack of direct comparative evidence. Despite their potential, organoids currently face several challenges that limit their therapeutic utility. These include poor vascularization, limited structural maturity, heterogeneous and non-standardized cellular composition, and difficulties associated with transplantation, such as larger grafts and an increased surgical risk. Furthermore, organoids often contain undifferentiated or off-target cells, which may increase the risk of tumor formation or unwanted tissue development. From an ethical standpoint, while concerns around cell transplantation have subsided, the use of brain organoids, especially those mimicking advanced neural features, continues to provoke ethical debate and requires further societal and scientific deliberation [157]. Based on these considerations, before translating into clinical applications, more studies are required to determine the optimal dose and timing for organoid transplantation. Moreover, the development of reliable quality control and manufacturing methods and the demonstration of organoids’ potential advantages compared to other strategies need to be established.

## Figures and Tables

**Table 1:** Search terms used in PubMed and Embase databases.

PubMed	Embase
(“Organoids” [Mesh] OR spheroid OR assembloid) AND (Brain [Mesh] OR cerebr* OR cortical OR forebrain OR hindbrain OR midbrain OR striatal OR thalam* OR hypothalam* OR cerebel* OR ganglionic eminence [Mesh]) AND (“Transplantation” [Mesh] OR “Transplants” [Mesh] OR implant* OR graft)	((Brain or cerebr* or cortical or forebrain or hindbrain or midbrain or striatal or thalam* or hypothalam* or cerebel* or ganglionic eminence) organoid OR ((brain or cerebr* or cerebel* or cortical*) spheroid) OR brain assembloid) AND (transplant* or implant* or graft)

**Table 2:** Summary of brain organoid transplantation articles.

Proof-of-Principle									
Transplanted Organoids	Cell Type Derived	Extracellular Scaffold During Organoid Culture	Receiver Age or Size	Immunosuppressive Treatment	Brain Injury Site	Transplant Timing	Evaluation Time Post Implantation	Main Findings and Measurements	References
Cerebral organoids 40–50 DIV	hESCs	Matrigel	NOD/SCID mice 5 to 6 weeks old N = 3–7	None	Aspirative lesion Retrosplenial cortex	Immediately after injury	0.5–8 months	Increased neuronal differentiation and maturation Developed functional synaptic connectivity and neuronal activity between grafted and host brain	Mansour et al., 2018 [12]
Cerebral organoids 47–48 DIV (or dissociated neural progenitor cells)	hESCs hiPSCs	Matrigel	CD1 mice P8–P10	None	Knife stab lesion Frontoparietal cortex	Immediately after injury	2 and 4 weeks	Increased cell survival and neuronal differentiation compared to dissociated cells transplantation Robust vascularization from host	Daviaud et al., 2018 [13]
Dorsal forebrain organoids 42 and 70 DIV Cut into 1 mm pieces Transplant 3, 5, or 11 pieces	hESCs	ND	SCID mice 7 days old or 6 weeks old	None	Aspirative lesion Bilateral frontal and parietal cortices	No delay or 1 week after injury	12 weeks	Extended axons along the host corticospinal tract 42 DIV organoids caused graft overgrowth after transplantation 1-week delayed transplantation group had greater axonal extensions than no delay group	Kitahara et al., 2020 [25]
			Cynomolgus monkeys 3 years old	Tacrolimus hydrate	Aspirative lesion Precentral cortex	1 week after injury			
Cerebral organoids × 3–5 40–50 DIV 150–250 μm, small controlled size (or dissociated cells)	hESCs hiPSCs	ND	SCID mice 6 to 8 weeks old	None	No additional injury Medial prefrontal cortex	N/A	1–5 months	Grafts survived Extended projections to basal brain regions within 1 month Generated human glutamatergic neurons with electrophysiological maturity Functionally integrated into host neural circuits by forming bidirectional synaptic connections Increase the startle fear response of host compared to control group	Dong et al., 2021 [26]
Dorsal forebrain organoids 30–60 DIV	hiPSCs (control, TS)	ND	Athymic (FOXN1^− /−^) newborn rat 3 to 7 days old	None	No additional injury S1 cortex	N/A	3–8 months	Transplanted cells displayed more complex morphological, synaptic, and intrinsic membrane properties than in vitro counterparts Transplanted organoids received thalamocortical and corticocortical inputs, and can produce sensory responses in grafted cells Grafts mature and engage host circuits that control behavior	Revah et al., 2022 [19]
Dorsal forebrain organoids 49–63 DIV Co-implanted with microelectrode array	hiPSCs	ND	NOD-SCID mice Female 8–12 weeks old	None	Aspirative lesion Left retrosplenial cortex	Immediately after injury	8–11 weeks	Visual stimuli could evoke electrophysiological responses in the grafted organoids	Wilson et al., 2022 [27]
Dorsal forebrain organoids 80–88 DIV Pretreat with Nec-1 (or dissociated cells)	hESCs hiPSCs	Matrigel	Long Evans rats Male 8–12 weeks old 250–300 g	Cyclosporine A	Aspirative lesion Visual cortex	Immediately after injury	3 months	Synaptically connected to the host retina and visual system; respond to host visual stimulation	Jgamadze et al., 2023 [20]
Dorsal forebrain organoids 40 DIV ES pretreated, 400 mV, 250 Hz Cut into 1 mm-diameter pieces	hESCs	ND	NOD-SCID mice 4 to 6 weeks old	None	Aspirative lesion S1 cortex	Immediately after injury	months	ES treatment could facilitate the development of cortical organoids ES pretreated organoid grafts had higher viability and maturity, longer axonal projections, and more vascularization ES pretreated organoid grafts could improve functional integration to the host	Li et al., 2024 [28]
**Therapeutic-Traumatic Brain Injury**									
**Transplanted Organoids**	**Cell Type Derived**	**Extracellular Scaffold**	**Receiver** **Age or Size**	**Immunosuppressive Treatment**	**Brain Injury** **Site**	**Transplant Timing**	**Evaluation Time** **Post Implantation**	**Main findings and Measurements**	**References**
Cerebral organoids 55 and 85 DIV	hESCs	Matrigel	Sprague–Dawley rats Male 250 ± 30 g	Cyclosporine A	Biopsy punch lesion Right motor cortex	Immediately after injury	8 weeks	Increased neurogenesis and cell survival Improved motor function and reduced brain injury 55 DIV > 80 DIV: More grafted cells survival better proliferation and differentiation	Wang Z. et al., 2020 [14]
Cerebral organoids 58 DIV	hESCs	Matrigel	SCID mice Male 8 weeks old	None	Impactor lesion Left parietal cortex	1 week after injury	70 days	Grafts mature and differentiation Filling brain lesion area Improved memory and learning ability Reduced glial scar Electrophysiological activities of implanted cells	Bao et al., 2021 [15]
Cerebral organoids 56 DIV	hESCs	Matrigel	C57BL/6J mice Male 7 to 8 weeks old	Cyclosporine A	Biopsy punch lesion Retrosplenial cortex	1 week after injury	7 and 14 days	Organoid transplantation could Reduce cell death Induce neurogenesis Promote vascularization Improve performance in novel objection recognition test	Kim et al., 2022 [29]
Dorsal forebrain organoids 40 DIV Stimulated via OBCIs in vivo, 50–70 Hz, 50 μA Cut into 1 mm-diameter pieces	hESCs	ND	NOD-SCID mice Male 4 to 6 weeks old	None	Aspirative lesion S1 cortex	Immediately after injury	40–180 days	OBCI stimulation could promote organoid development, vascularization, synaptic connection, electrophysiological maturation OBCI treatment may facilitate the recovery of the pain response	Hu et al., 2024 [30]
**Therapeutic-Stroke**									
**Transplanted Organoids**	**Cell type Derived**	**Extracellular Scaffold**	**Receiver** **Age or Size**	**Immunosuppressive Treatment**	**Brain Injury** **Site**	**Transplant Timing**	**Evaluation Time** **Post Implantation**	**Main Findings and Measurements**	**References**
Cerebral organoids × 2 55 DIV	hESCs	Matrigel	Sprague–Dawley rats Male 250 ± 30 g	Cyclosporine A	MCAO Left motor cortex	6 h, 24 h, or 7 days after MCAO	4 weeks	Reduced cerebral infarct volume Enhance axonal regeneration and synaptic reconstruction 6 h > 24 h > 7 days after MCAO: reduced infarct volume and improved neurological function	Wang S. N. et al., 2020 [16]
Cerebral organoids × 1–3 50 DIV 600 μm ~70,000 cells each (also dissociated cells)	hESCs hiPSCs	ND	NOD-SCID mice Male 7 to 8 weeks old	None	Photothrombotic stroke Forelimb motor cortex Transplanted to junction of the infarct core and the peri-infarct zone	1 week post-stroke	180 days	Differentiated into target neurons Repaired infarcted tissue Sent axons to distant brain targets Integrated into the host neural circuit Eliminated sensorimotor defect behaviors	Cao et al., 2023 [17]
Brain organoids resembling the MGE domain × 3 50 DIV 600 μm in diameter ~200,000 cells total	hiPSCs	ND	NOD-SCID mice Male 7 to 8 weeks old	None	Photothrombotic stroke Forelimb motor cortex Transplanted to junction of the infarct core and the peri-infarct zone	1 week post-stroke	150 days	Differentiated into GABAergic interneurons Functionally restored the sensorimotor deficits	Cao et al., 2023 [31]
**Therapeutic-Parkinson’s Disease**									
**Transplanted Organoids**	**Cell Type Derived**	**Extracellular Scaffold**	**Receiver** **Age or Size**	**Immunosuppressive Treatment**	**Brain Injury** **Site**	**Transplant Timing**	**Evaluation Time** **Post Implantation**	**Main Findings and Measurements**	**References**
Midbrain organoids 10, 15, 25 DIV ~400,000 cells each Cut into small pieces to be able to pass through a 10 μL pipette tip	hiPSCs	Matrigel	SCID mice 8 to 12 weeks old	None	6-OHDA-lesioned PD model Right striatum	4-week post-6-OHDA treatment	6, 12, and 16 weeks	Led to a reversal of motor function and establishment of bidirectional connections with natural brain target regions No incidence of tumor formation or graft overgrowth The 15 DIV hMOs were the most appropriate stage rather than 10 or 25 DIV ones	Zheng et al., 2023 [32]
**Vascularization**									
**Transplanted Organoids**	**Cell Type Derived**	**Extracellular Scaffold**	**Receiver** **Age or Size**	**Immunosuppressive Treatment**	**Brain Injury** **Site**	**Transplant Timing**	**Evaluation Time** **Post Implantation**	**Main Findings and Measurements**	**References**
Cerebral organoids with iPSC-derived ECs 54 DIV	hiPSCs	Matrigel	NOD/SCID gamma mice Male 2 months old	None	Direct removal of brain tissue Location not specified.	Immediately after injury	2 weeks	Vascularization of brain organoids with a donor’s own iPSC-derived ECs	Pham, M. T. et al., 2018 [21]
Dorsal forebrain organoids with human umbilical vein endothelial cells 60 DIV	hESCs hiPSCs	Matrigel	NOD-SCID mice 8 weeks old	None	Aspirative lesion S1 cortex	Immediately after injury	3 days to 2 months	Constructed functional human-mouse blood vessels in the grafts that promoted cell survival in the grafts	Shi, Y. et al., 2020 [22]
**Disease Modeling and Cell Study Platform In Vivo**									
**Transplanted Organoids**	**Cell Type Derived**	**Extracellular Scaffold**	**Receiver** **Age or Size**	**Immunosuppressive Treatment**	**Brain Injury** **Site**	**Transplant Timing**	**Evaluation Time** **Post Implantation**	**Main Findings and Measurements**	**References**
Cerebral organoids × 3 7 DIV (or dissociated cells)	hiPSCs	Matrigel	SCID mice Female 4 to 5 weeks old	None	No additional injury Corpus striatum	N/A	2 months	Grafted organoids developed more pericyte-like and choroid plexus cells Grafted organoids had lower levels of cellular stress and apoptosis	Huang et al., 2022 [33]
Dorsal forebrain organoids 52 DIV with or without stem cell-derived erythromyeloid progenitors coculture for 10–14 days	hESCs hiPSCs (including ASD)	Matrigel	NOD-SCID mice Mainly female 6 to 10 weeks old	None	Aspirative cavity Retrosplenial cortex	Immediately after injury	6–12 weeks	Organoid-resident hMGs gain human-specific transcriptomic signatures that closely resemble their in vivo counterparts within organoid hMGs could react to local injuries, and respond to systemic inflammatory cues in the human-brain-like environment under physiological and pathological conditions in vivo	Schafer et al., 2023 [34]
Glia-enriched cerebral organoids 56–70 DIV	hESCs hiPSCs	Matrigel	NOD-SCID mice 6 to 12 weeks old	None	Aspirative cavity Retrosplenial cortex	Immediately after injury	5, 6, and 8 months	Glia-enriched organoids could generate a diverse repertoire of cortical neurons and anatomical subclasses of human astrocytes The subpopulation of astrocytes can rapidly activate proinflammatory pathways upon cytokine stimulation	Wang et al., 2024 [35]
Dorsal forebrain organoids 30–60 DIV	hiPSCs (control, TS)	ND	Athymic (FOXN1^− /−^) newborn rat 3 to 7 days old	None	No additional injury S1 cortex	N/A	14 days	After antisense oligonucleotide treatment: Expression of CACNA1C exon 8A in the transplanted organoids would be reduced Post-depolarization residual calcium of TS neuron could be normalized Dendritic morphology of TS neurons could be corrected	Chen et al., 2024 [36]
Cerebral organoids 42 DIV 150–250 μm in diameter ~50,000 cells (5 organoids) each site	hESCs hiPSCs	ND	SCID mice Male 6 to 8 weeks old	None	No additional injury Prefrontal cortex or hippocampus	N/A	2–4 months	Grafted cells undergo neural development at 2 mpt and then glial development at 4 mpt Received host niche factor regulation after transplantation, resulting in the alignment of grafted cell fate with implanted brain regions	Xu et al., 2024 [37]
Dorsal forebrain organoids 50 DIV LIUS pretreated in vitro, or in vivo LIUS modulating Cut into 0.5 mm-diameter pieces	hESCs (control, ASPM mutant)	ND	NOD-SCID mice 4 to 6 weeks old	None	Aspirative lesion S1 cortex	Immediately after injury	2–5 months	LIUS treatment could enhance the following features: Neural progenitor cell proliferation and neuronal maturation Axonal projections Vascularization Electrophysiological activity Functional integration Rescuing microcephaly deficits	Li et al., 2024 [38]

6-OHDA, 6-hydroxydopamine; ASD, autism spectrum disorder; DIV, days in vitro; ECs, endothelial cells; ES, electrical stimulation; hESCs, human embryonic stem cells; hiPSCs, human-induced pluripotent stem cells; hMG, human microglia; hMOs, human midbrain organoids; MCAO, middle cerebral artery occlusion; MGE, medial ganglionic eminence; mpt, months post-transplantation; N/A, not applicable; ND, not described; NOD, nonobese diabetic; OBCIs, organoid–brain–computer interfaces; PD, Parkinson’s disease; SCID, severe combined immunodeficiency; TS, Timothy syndrome.

**Table 3:** Risk of bias assessment with SYRCLE’s tool.

Proof-of-Principle										
	Sequence Generation	Baseline Characteristics	Allocation Concealment	Random Housing	Performance Blinding	Random Outcome Assessment	Detection Blinding	Incomplete Outcome Data	Selective Outcome Reporting	Other Sources of Bias
Mansour et al., 2018 [12]	Unclear Risk	Unclear Risk	Unclear Risk	Unclear Risk	Unclear Risk	High Risk	High Risk	Low Risk	Low Risk	Low Risk
Daviaud et al., 2018 [13]	Unclear Risk	Unclear Risk	Unclear Risk	Unclear Risk	Unclear Risk	Unclear Risk	Unclear Risk	Unclear Risk	Low Risk	Low Risk
Kitahara et al., 2020 [25]	Unclear Risk	Unclear Risk	Unclear Risk	Unclear Risk	Unclear Risk	Unclear Risk	Unclear Risk	Unclear Risk	Low Risk	Low Risk
Dong et al., 2021 [26]	Low Risk	Unclear Risk	Low Risk	Unclear Risk	Low Risk	Low Risk	Low Risk	Low Risk	Low Risk	Low Risk
Revah et al., 2022 [19]	Unclear Risk	Unclear Risk	Unclear Risk	Unclear Risk	Low Risk	Unclear Risk	Low Risk	Low Risk	Low Risk	Low Risk
Wilson et al., 2022 [27]	Unclear Risk	Unclear Risk	Unclear Risk	Unclear Risk	Unclear Risk	Unclear Risk	Unclear Risk	Low Risk	Low Risk	Low Risk
Jgamadze et al., 2023 [20]	Unclear Risk	Unclear Risk	Unclear Risk	Unclear Risk	Unclear Risk	Unclear Risk	Unclear Risk	Low Risk	Low Risk	Low Risk
Li et al., 2024 [28]	Low Risk *	Unclear Risk	Low Risk	Unclear Risk	Low Risk	Low Risk	Low Risk	Low Risk	Low Risk	Low Risk
**Therapeutic—Traumatic Brain Injury**										
	**Sequence Generation**	**Baseline Characteristics**	**Allocation Concealment**	**Random Housing**	**Performance Blinding**	**Random Outcome Assessment**	**Detection Blinding**	**Incomplete Outcome Data**	**Selective Outcome Reporting**	**Other Sources of Bias**
Wang Z. et al., 2020 [14]	Low Risk *	Low Risk	Low Risk	Unclear Risk	Low Risk	Low Risk	Low Risk	Low Risk	Low Risk	Low Risk
Bao et al., 2021 [15]	Low Risk *	Unclear Risk	Low Risk	Unclear Risk	Unclear Risk	Unclear risk	Unclear risk	Low Risk	Low Risk	Low Risk
Kim et al., 2022 [29]	Low Risk *	Unclear Risk	Low Risk	Unclear Risk	Unclear Risk	Unclear Risk	Low Risk	Low Risk	Low Risk	Low Risk
Hu et al., 2024 [30]	Low Risk *	Unclear Risk	Low Risk	Unclear Risk	Low Risk	Low Risk	Low Risk	Unclear risk	Low Risk	Low Risk
**Therapeutic—Stroke**										
	**Sequence Generation**	**Baseline Characteristics**	**Allocation Concealment**	**Random Housing**	**Performance Blinding**	**Random Outcome Assessment**	**Detection Blinding**	**Incomplete Outcome Data**	**Selective Outcome Reporting**	**Other Sources of Bias**
Wang S. N. et al., 2020 [16]	Low Risk *	Low Risk	Low Risk	Unclear Risk	Low Risk	Low Risk	Unclear risk	Low Risk	Low Risk	Low Risk
Cao et al., 2023 [17]	Unclear Risk	Low Risk	Unclear risk	Unclear Risk	Low Risk	Unclear risk	Low Risk	Low Risk	Low Risk	Low Risk
Cao et al., 2023 [31]	Low Risk *	Low Risk	Low Risk	Unclear Risk	Low Risk	Low Risk	Unclear risk	Low Risk	Low Risk	Low Risk
**Therapeutic—Parkinson’s Disease**										
	**Sequence Generation**	**Baseline Characteristics**	**Allocation Concealment**	**Random Housing**	**Performance Blinding**	**Random Outcome Assessment**	**Detection Blinding**	**Incomplete Outcome Data**	**Selective Outcome Reporting**	**Other Sources of Bias**
Zheng et al., 2023 [32]	Unclear Risk	Low Risk	Unclear Risk	Unclear Risk	Unclear Risk	Unclear risk	Unclear risk	Unclear Risk	Low Risk	Low Risk
**Vascularization**										
	**Sequence Generation**	**Baseline Characteristics**	**Allocation Concealment**	**Random Housing**	**Performance Blinding**	**Random Outcome Assessment**	**Detection Blinding**	**Incomplete Outcome Data**	**Selective Outcome Reporting**	**Other Sources of Bias**
Pham, M. T. et al., 2018 [21]	Unclear Risk	Unclear Risk	Unclear Risk	Unclear Risk	Unclear Risk	Unclear risk	Unclear risk	Low Risk	Low Risk	High Risk
Shi, Y. et al., 2020 [22]	Unclear Risk	Unclear Risk	Unclear Risk	Unclear Risk	Unclear Risk	Unclear risk	Unclear risk	Low Risk	Low Risk	Low Risk
**Disease Modeling and Cell Study Platform In Vivo**										
	**Sequence Generation**	**Baseline Characteristics**	**Allocation Concealment**	**Random Housing**	**Performance Blinding**	**Random Outcome Assessment**	**Detection Blinding**	**Incomplete Outcome Data**	**Selective Outcome Reporting**	**Other Sources of Bias**
Huang et al. 2022 [33]	Unclear Risk	Unclear Risk	Unclear Risk	Unclear Risk	Unclear Risk	Unclear risk	Unclear risk	Low Risk	Low Risk	Low Risk
Schafer et al., 2023 [34]	Unclear Risk	Unclear Risk	Unclear Risk	Unclear Risk	Unclear Risk	Unclear risk	Unclear risk	Low Risk	Low Risk	Low Risk
Wang et al., 2024 [35]	Low Risk *	Unclear Risk	Low Risk	Unclear Risk	Unclear Risk	Low Risk	Unclear risk	Low Risk	Low Risk	Low Risk
Chen et al., 2024 [36]	Unclear Risk	Unclear Risk	Unclear Risk	Unclear Risk	High Risk	Unclear risk	Unclear risk	Low Risk	Low Risk	Low Risk
Xu et al., 2024 [37]	Low Risk *	Unclear Risk	Low Risk	Unclear Risk	Unclear Risk	Low Risk	Unclear risk	Low Risk	Low Risk	Low Risk
Li et al., 2024 [38]	Low Risk *	Unclear Risk	Low Risk	Unclear Risk	Unclear Risk	Low Risk	Low Risk	Low Risk	Low Risk	Low Risk

* Randomized, but the method of randomization was not described.

**Table 4:** Keynote summary of transplantation procedures.

Proof-of-Principle					
	Animal	Location	Lesion Created Method and Size or Delivery Method	Fix and Cover	Immunosuppressive Treatment
Mansour et al., 2018 [12]	SCID mice	Retrosplenial cortex	Aspirative lesion	Covered by 5 mm cover slip sealed with adhesive glue	None
Daviaud et al., 2018 [13]	CD 1 mice (newborn)	Frontoparietal cortex	Knife stab lesion 1 mm^3^ cavity	Covered by Bone flap sealed with fibrin glue	None
Kitahara et al., 2020 [25]	SCID mice (newborn and adult)	Bilateral frontal and parietal cortices	Aspirative lesion 1 mm diameter and 1 mm depth	Returned the hinged bone flap	None
	Cynomolgus monkeys	Precentral cortex	Lesion created method ND 2 mm cavity	ND	Tacrolimus hydrate
Dong et al., 2021 [26]	SCID mice	Medial prefrontal cortex	No additional lesion Injection using a glass micropipette	ND	None
Revah et al., 2022 [19]	Athymic rats (newborn)	S1 cortex	No additional lesion Direct injection with Hamilton syringe and 23-gauge needle	ND	None
Wilson et al., 2022 [27]	SCID mice	Left retrosplenial cortex	Aspirative lesion 1 mm diameter	Covered by a glass plug with 16 channels microelectrode array sealed with dental adhesive	None
Jgamadze et al., 2023 [20]	LE rats	Right visual cortex	Aspirative lesion 2–2.5 mm diameter and depth to the corpus callosum	Covered by PDMS cranioplasty cap sealed with bone cement PMMA	Cyclosporine A
Li et al., 2024 [28]	SCID mice	S1 cortex	Aspirative cavity 0.5 mm in diameter, 0.8 mm in depth	Returned the hinged bone flap	None
**Therapeutic—Traumatic Brain Injury**					
	**Animal**	**Location**	**Lesion Created Method and Size or Delivery Method**	**Fix and Cover**	**Immunosuppressive Treatment**
Wang Z. et al., 2020 [14]	SD rats	Right motor cortex	Biopsy punch cavity 3 mm diameter and 2 mm depth	Sealed with piece of skull and bone wax.	Cyclosporine A
Bao et al., 2021 [15]	SCID mice	Left parietal cortex	By impactor tip 1.4 mm depth	ND	None
Kim et al., 2022 [29]	C57BL/6J mice	Retrosplenial cortex above the hippocampus	Biopsy punch lesion 2 mm cavity	Returned the hinged bone flap and sealed with fibrin glue	Cyclosporine A
Hu et al., 2024 [30]	SCID mice	S1 cortex	Aspirative cavity 0.5 mm in diameter, 0.8 mm in depth	Returned the hinged bone flap	None
**Therapeutic—Stroke**					
	**Animal**	**Model and Location**	**Lesion Created Method and Size or Delivery Method**	**Fix and Cover**	**Immunosuppressive Treatment**
Wang S. N. et al., 2020 [16]	SD rats	MCAO Left motor cortex	Biopsy punch 3 mm in diameter, 2 mm in depth	Covered by piece of excised skull sealed with the bone wax	Cyclosporine A
Cao et al., 2023 [17]	SCID mice	Photothrombotic stroke Forelimb motor cortex Transplanted to junction of the infarct core and the peri-infarct zone	Microinjection with borosilicate glass capillary	ND	None
Cao et al., 2023 [31]	SCID mice	Photothrombotic stroke Forelimb motor corte Transplanted to junction of the infarct core and the peri-infarct zone	Microinjection with borosilicate glass capillary	ND	None
**Therapeutic—Parkinson’s Disease**					
	**Animal**	**Model and Location**	**Lesion Created Method and Size or Delivery Method**	**Fix and Cover**	**Immunosuppressive Treatment**
Zheng et al., 2023 [32]	SCID mice	6-OHDA-lesioned PD model Right striatum	Injected with a microinjector	ND	None
**Vascularization**					
	**Animal**	**Location**	**Lesion Created Method and Size or Delivery Method**	**Fix and Cover**	**Immunosuppressive Treatment**
Pham, M. T. et al., 2018 [21]	SCID mice	Location not specified	Lesion created method ND 2 mm × 2 mm × 2 mm	ND	None
Shi, Y. et al., 2020 [22]	SCID mice	S1 cortex	Aspirative lesion	3% low-melting agarose and adhesive glue to fix grafts	None
**Disease modeling and cell study platform in vivo**					
	**Animal**	**Location and Cavity size**	**Lesion Created Method and Size or Delivery Method**	**Fix and Cover**	**Immunosuppressive Treatment**
Huang et al. 2022 [33]	SCID mice	Corpus striatum	No additional lesion Direct injection with Hamilton syringe and 22-gauge needle	ND	None
Schafer et al., 2023 [34]	SCID mice	Retrosplenial cortex On the pial vessels caudal to the hippocampus	Aspirative cavity	Covered by custom titanium head plate fixed with dental cement	None
Wang et al., 2024 [35]	SCID mice	Retrosplenial cortex Forelimb motor cortex Transplanted to junction of the infarct core and the peri-infarct zone	Aspirative cavity	Covered by 5 mm cover slip sealed with adhesive glue and dental cement for the wound	None
Chen et al., 2024 [36]	Athymic rats (newborn)	S1 cortex	No additional lesion Direct injection with Hamilton syringe and 23-gauge needle	ND	None
Xu et al., 2024 [37]	SCID mice	Prefrontal cortex or hippocampus	No additional lesion Direct injection with glass electrodes	ND	None
Li et al., 2024 [38]	SCID mice	S1 cortex	Aspirative cavity 0.5 mm in diameter, 0.8 mm in depth	Returned the hinged bone flap	None

6-OHDA, 6-hydroxydopamine; LE, Long Evans; MCAO, middle cerebral artery occlusion; ND, not described; PD, Parkinson’s disease; PDMS, Polydimethylsiloxane; PMMA, polymethylmethacrylate; SCID, severe combined immunodeficiency; SD, Sprague–Dawley.

**Table 5:** Summary of major outcome measurements.

Proof-of-Principle									
	Cell Survival	Neuronal Differentiation	Axonal Projection	Electrophysiology	In vivo Microscopic Imaging	Optogenetics	Vascularization	Behavior Tests	Others
Mansour et al., 2018 [12]	+	+	ND	+	+	+	+	Barnes maze test −	
Daviaud et al., 2018 [13]	+	+	ND	ND	ND	ND	+	ND	
Kitahara et al., 2020 [25]	+	+	+	ND	ND	ND	+	ND	MRI
Dong et al., 2021 [26]	+	+	ND	+	ND	+	ND	Open field test − Fear conditioning +	
Revah et al., 2022 [19]	+	+	+	+	+	+	+	Optogenetic behavioral assay + Open field test − Novel object recognition − Fear conditioning −	MRI EEG snRNA-seq Neuron morphology
Wilson et al., 2022 [27]	+	+	ND	+	+	ND	+	ND	Visual stimulation
Jgamadze et al., 2023 [20]	+	+	+	+	ND	ND	+	ND	Rabies virus retrograde tracing HSV anterograde tracing Visual stimulation
Li et al., 2024 [28]	+	+	+	+	+	-	+	Pain behavioral tests + Open field test − Novel object recognition test −	snRNA-seq Western blot for CAMKII-PKA-CREB pathway Neural signals analysis Functional connectivity map
**Therapeutic—Traumatic Brain Injury**									
	**Cell Survival**	**Neuronal Differentiation**	**Axonal Projection**	**Electrophysiology**	**In Vivo Microscopic Imaging**	**Optogenetics**	**Vascularization**	**Behavior Tests**	**Others**
Wang Z. et al., 2020 [14]	+	+	ND	ND	ND	ND	+	mNSS evaluation + Beam walking test +	Western blot for neurotrophic factors
Bao et al., 2021 [15]	+	+	ND	+	ND	ND	+	Morris water maze test +/− * Passive avoidance assay +	MRI
Kim et al., 2022 [29]	+	+	ND	ND	ND	ND	+	Novel object recognition test +	Cytokine and chemokine profile
Hu et al., 2024 [30]	+	+	+	+	ND	ND	+	Pain behavioral tests + Open field test − Novel object recognition test −	Neural signals analysis
**Therapeutic—Stroke**									
	**Cell Survival**	**Neuronal Differentiation**	**Axonal Projection**	**Electrophysiology**	**In Vivo Microscopic Imaging**	**Optogenetics**	**Vascularization**	**Behavior Tests**	**Others**
Wang S. N. et al., 2020 [16]	+	+	+	ND	ND	ND	+	mNSS evaluation + Beam walking test +	
Cao et al., 2023 [17]	+	+	+	+	ND	+	ND	Cylinder test + Grid-walking test + Adhesive removal test +	
Cao et al., 2023 [31]	+	+	ND	+	+	ND	ND	Cylinder test + Grid-walking test + Adhesive removal test +	
**Therapeutic—Parkinson’s Disease**									
	**Cell Survival**	**Neuronal Differentiation**	**Axonal Projection**	**Electrophysiology**	**In vivo Microscopic Imaging**	**Optogenetics**	**Vascularization**	**Behavior Tests**	**Others**
Zheng et al., 2023 [32]	+	+	+	+	ND	ND	ND	APO-induced rotation test + Rotarod test + Open field test +	mRNA expressions assay HPLC analysis for neurotransmitters Rabies virus retrograde tracing
**Vascularization**									
	**Cell Survival**	**Neuronal Differentiation**	**Axonal Projection**	**Electrophysiology**	**In Vivo Microscopic Imaging**	**Optogenetics**	**Vascularization**	**Behavior Tests**	**Others**
Pham, M. T. et al., 2018 [21]	+	ND	ND	ND	ND	ND	+, graft	ND	
Shi, Y. et al., 2020 [22]	+	+	ND	+	+	ND	+, graft	ND	snRNA-seq and cell type mapping
**Disease Modeling and Cell Study Platform In Vivo**									
	**Cell Survival**	**Neuronal Differentiation**	**Axonal Projection**	**Electrophysiology**	**In Vivo Microscopic Imaging**	**Optogenetics**	**Vascularization**	**Behavior Tests**	**Others**
Huang et al. 2022 [33]	+	+	ND	ND	ND	ND	+		snRNA-seq
Schafer et al., 2023 [34]	+, microglia	+, microglia	ND	ND	+	ND	ND	ND	snRNA-seq
Wang et al., 2024 [35]	+, astrocytes	+, astrocytes	ND	+	ND	ND	+	ND	snRNA-seq Mitochondrial morphological and function analysis for astrocytes Glutamate uptake assay for astrocytes
Chen et al., 2024 [36]	+	ND	ND	ND	+	ND	ND	ND	MRI Neuron morphology
Xu et al., 2024 [37]	+	+	ND	ND	+	ND	+	ND	snRNA-seq Glutamate clearance assay for astrocytes Morphology analysis after adding neurotransmitters for astrocytes
Li et al., 2024 [38]	+	+	+	+	ND	ND	+	Pain behavioral tests +	snRNA-seq Western blot for YAP Neural signals analysis Functional connectivity map

+ means positive findings. − means no change compared to control group. * Improved in spatial learning and memory, no change in swimming speed. APO, Apomorphine; EEG, electroencephalogram; HPLC, high-performance liquid chromatography; HSV, herpes simplex virus; mNSSs, modified neurological severity scores; ND, not described; MRI, magnetic resonance imaging; snRNA-seq, single-nucleus RNA sequencing.

**Table 6:** Comparison of conventional culture and brain organoid-on-chip.

	Conventional Culture	Brain Organoid-on-Chip
**Organoid**		
Necrotic core	Significant	Minimized
Neurogenesis and corticogenesis	Fair	Enhanced
Electrophysiological activity	Fair	Enhanced
Heterogeneity	High	Relatively lower
Batch variability	High	Relatively lower
**System**		
Nutrients and gases exchange	Poor	Good
Precise microenvironments monitor and control	Hard	Feasible
Manual intervention/automatic level	Much/low	Less/high
Technical requirements	Relatively lower	High
System maintenance	Relatively easier	Complicated
Cost	Fair	Relatively higher in developing phase; may be lower in mature batch processing with precise control

## Data Availability

All data generated or analyzed during this study are included in this published article. Figure 3 was created in BioRender. Shen, Y. (2025) https://BioRender.com/hbzxbsm, accessed on 7 July 2025.
